# Anti-miR-518d-5p overcomes liver tumor cell death resistance through mitochondrial activity

**DOI:** 10.1038/s41419-021-03827-0

**Published:** 2021-05-28

**Authors:** Pablo Fernández-Tussy, Rubén Rodríguez-Agudo, David Fernández-Ramos, Lucía Barbier-Torres, Imanol Zubiete-Franco, Sergio López de Davalillo, Elisa Herraez, Naroa Goikoetxea-Usandizaga, Sofia Lachiondo-Ortega, Jorge Simón, Fernando Lopitz-Otsoa, Virginia Gutiérrez-de Juan, Misti V. McCain, Maria J. Perugorria, Jon Mabe, Nicolás Navasa, Cecilia M. P. Rodrigues, Isabel Fabregat, Loreto Boix, Victor Sapena, Juan Anguita, Shelly C. Lu, José M. Mato, Jesus M. Banales, Erica Villa, Helen L. Reeves, Jordi Bruix, Maria Reig, Jose J. G. Marin, Teresa C. Delgado, María L. Martínez-Chantar

**Affiliations:** 1 Liver Disease Laboratory, Precision Medicine and Metabolism Laboratory, Center for Cooperative Research in Biosciences (CIC bioGUNE), Basque Research and Technology Alliance (BRTA), Derio, Spain; 2 Centro de Investigación Biomédica en Red de Enfermedades Hepáticas y Digestivas (CIBERehd), Instituto de Salud Carlos III, Madrid, Spain; 3 Experimental Hepatology and Drug Targeting (HEVEPHARM), University of Salamanca, IBSAL, Salamanca, Spain; 4 Northern Institute for Cancer Research, The Medical School, Newcastle University, Newcastle upon Tyne, UK; 5 Department of Liver and Gastrointestinal Diseases, Biodonostia Research Institute, Donostia University Hospital, University of the Basque Country (UPV/EHU), San Sebastian, Spain; 6 IKERBASQUE, Basque Foundation for Science, Bilbao, Spain; 7 Electronics and Communications Unit, IK4-Tekniker, Eibar, Spain; 8 Inflammation and Macrophage Plasticity, CIC bioGUNE, Derio, Bizkaia Spain; 9 Research Institute for Medicines (iMed.ULisboa), Faculty of Pharmacy, Universidade de Lisboa, Lisbon, Portugal; 10 TGF-β and Cancer Group, Oncobell Program, Bellvitge Biomedical Research Institute (IDIBELL) and University of Barcelona, Barcelona, Spain; 11 Barcelona-Clínic Liver Cancer Group, Liver Unit, Institut d’Investigacions Biomèdiques August Pi I Sunyer,Hospital Clínic, Universitat de Barcelona, Barcelona, Catalonia Spain; 12 Karsh Division of Gastroenterology and Hepatology, Cedars-Sinai Medical Center, Los Angeles, CA USA; 13 Department of Gastroenterology, Azienda Ospedaliero-Universitaria and University of Modena and Reggio Emilia, Modena, Italy; 14 Hepatopancreatobiliary Multidisciplinary Team, Freeman Hospital, Freeman Road, Newcastle upon Tyne NHS Hospitals Foundation Trust, Newcastle upon Tyne, NE7 7DN UK

**Keywords:** Cancer epigenetics, Apoptosis, Biomarkers

## Abstract

Dysregulation of miRNAs is a hallmark of cancer, modulating oncogenes, tumor suppressors, and drug responsiveness. The multi-kinase inhibitor sorafenib is one of the first-line drugs for advanced hepatocellular carcinoma (HCC), although the outcome for treated patients is heterogeneous. The identification of predictive biomarkers and targets of sorafenib efficacy are sorely needed. Thus, selected top upregulated miRNAs from the C19MC cluster were analyzed in different hepatoma cell lines compared to immortalized liver human cells, THLE-2 as control. MiR-518d-5p showed the most consistent upregulation among them. Thus, miR-518d-5p was measured in liver tumor/non-tumor samples of two distinct cohorts of HCC patients (*n* = 16 and *n* = 20, respectively). Circulating miR-518d-5p was measured in an independent cohort of HCC patients receiving sorafenib treatment (*n* = 100), where miR-518d-5p was analyzed in relation to treatment duration and patient’s overall survival. In vitro and in vivo studies were performed in human hepatoma BCLC3 and Huh7 cells to analyze the effect of miR-518d-5p inhibition/overexpression during the response to sorafenib. Compared with healthy individuals, miR-518d-5p levels were higher in hepatic and serum samples from HCC patients (*n* = 16) and in an additional cohort of tumor/non-tumor paired samples (*n* = 20). MiR-518d-5p, through the inhibition of c-Jun and its mitochondrial target PUMA, desensitized human hepatoma cells and mouse xenograft to sorafenib-induced apoptosis. Finally, serum miR-518d-5p was assessed in 100 patients with HCC of different etiologies and BCLC-stage treated with sorafenib. In BCLC-C patients, higher serum miR-518d-5p at diagnosis was associated with shorter sorafenib treatment duration and survival. Hence, hepatic miR-518d-5p modulates sorafenib resistance in HCC through inhibition of c-Jun/PUMA-induced apoptosis. Circulating miR-518d-5p emerges as a potential lack of response biomarker to sorafenib in BCLC-C HCC patients.

## Introduction

Hepatocellular carcinoma (HCC) is the sixth most common cancer worldwide and the third leading cause of cancer-related death^1,2^. Despite recent progress in HCC treatment, long-term survival and the well-being of the patients remain poor. Sorafenib is one of the first-line systemic drugs for HCC treatment; although the survival benefit for patients is heterogeneous and depends on their baseline characteristics^3,4^. Recognizing signaling networks to improve and predict the activity of sorafenib and identifying the cause of drug resistance would be highly beneficial to guide a personalized HCC treatment and to determine the outcome of patients enrolled in clinical trials. Sorafenib inhibits several tyrosine kinase receptors, such as those triggering the RAF and MEK/ERK pathways^5^, the platelet-derived growth factor receptor (PDGFR), and vascular endothelial growth factor receptors (VEGFRs)^6^. Besides its antiangiogenic and antiproliferative effect, the pro-apoptotic effects of sorafenib have been reported to occur by different mechanisms, including downregulating of induced myeloid leukemia cell differentiation protein (*MCL-1*)^7^ and increasing the expression of the p53-upregulated modulator of apoptosis (PUMA, *BBC3*), which functions as a critical initiator of apoptosis in cancer cells^8,9^. Besides P53, *BBC3* transcription is also regulated by p53-independent pathways, including transcription factors, such as Foxo3A, C/EBP, E2F1, and c-Jun^8–11^. PUMA can promote apoptosis by inhibiting anti-apoptotic molecules of the Bcl2 family or by activating pro-apoptotic BAX-BAK proteins, leading to mitochondrial membrane permeabilization (MMP)^8,12,13^. A number of reports have described an essential role for the activation of the JNK/c-Jun pathway in the apoptotic and antiproliferative response to sorafenib, despite the controversy pertaining c-Jun function in cell cycle progression and HCC initiation^14–17^.

MiRNAs regulate biological processes including differentiation and metabolism, as well as cellular responses such as proliferation, apoptosis, and tumorigenesis^18,19^. In the liver, miRNA signatures have been associated with non-alcoholic fatty liver disease (NAFLD), cirrhosis, and liver cancer^20–27^. Different miRNAs have been associated with the development of resistance to chemotherapeutic agents and, notably, to sorafenib^28–33^. The biological significance and therapeutic potential of miRNAs in liver disease management are rapidly growing fields of research. In particular, miRNAs belonging to C19MC, the largest human miRNA cluster, have been reported to be overexpressed in certain HCC patients and to have protumorigenic and metastatic activity^34^. In this context, we aimed to identify miRNAs overexpressed in HCC that could contribute to tumor development and potentially regulate sorafenib resistance.

In this study, we report that hepatic and circulating miR-518d-5p levels are increased in human HCC. High circulating miR-518d-5p levels are associated with shorter sorafenib treatment duration and decreased overall survival in the Barcelona clinic for liver cancer (BCLC)-C HCC patients. Likewise, miR-518d-5p over-expression is associated with sorafenib resistance in human hepatoma cells and an experimental mouse xenograft model in vivo. *c-Jun* was identified as a miR-518d-5p target involved in the regulation of apoptosis following sorafenib administration. A complex regulatory network in the modulation of sorafenib response is presented, based on associations of miR-518d-5p, c-Jun, and PUMA, both in vitro and in preclinical animal models.

## Experimental procedures

### Human samples

All patients in the study gave informed consent to all clinical investigations, according to the principles embodied in the Declaration of Helsinki.

### miR-518d-5p expression in liver and serum of patients with HCC

miR-518d-5p levels were determined in liver tissue and paired serum samples from patients with HCC, cirrhosis, and healthy individuals of the Donostia University Hospital (San Sebastian, Spain). The clinical information of the study population is provided in Supplementary Table I and II. The project was approved by the Ethical Review Board of participating Institutions.

### miR-518d-5p expression in non-tumor (NT)/tumor (T) matched tissues from HCC patients

miR-518d-5p levels were determined in NT-T matched tissue samples obtained from ultrasonographic (US)-guided liver biopsy from 20 patients enrolled by Modena Hospital with liver cirrhosis and HCC detected during surveillance^35^. In brief, patients with compensated liver cirrhosis and HCC lesions detected under US surveillance underwent a dedicated imaging protocol (two computed tomography exams 6-weeks apart in absence of any other therapy to evaluate growth speed) and a US-guided liver biopsy. After the second computed scan, patients underwent therapy according to internationally accepted guidelines.

### miR-518d-5p levels in serum from HCC patients treated with sorafenib

Circulating miR-518d-5p levels were determined in two different cohorts of patients. The first study was approved by the Newcastle and North Tyneside Regional ethics committee, the Newcastle academic health partners bioresource (NAHPB), and the Newcastle upon Tyne NHS Foundation Trust Research and Development (R&D) department (References 10/H0906/41; NAHPB Project 48; REC 12/NE/0395; R&D 6579; Human Tissue Act license 12534). Serum samples of 16 patients were obtained from patients who received sorafenib between 1 February 2010 to 31 July 2017, who consented to the use of their surplus tissues initially obtained for diagnostic requirements for research purposes. All patients had a confirmed diagnosis and management of HCC according to standard European guidelines^36^. Patient demographics and clinicopathological information including age, sex, underlying liver disease etiology, combined BCLC stages, treatments administered, and response to treatment are summarized in Supplementary Table III (right column, Newcastle). Of the 16 patients studied, nine of them received sorafenib after TACE treatment, while seven received it as their first treatment. The median time to start sorafenib following the bio banked serum sample was 3.67 months (range 0.07–17.63). The median duration of treatment (400 mg twice daily) was 6.71 months (range 4.38–13.66 months). Survival data were recorded for all patients until 30 August 2018. Clinical parameters are summarized in Supplementary Table III (right column, Newcastle).

The second cohort of serum samples from 84 patients treated with sorafenib was from the Barcelona clinic liver cancer (BCLC) group, at Hospital Clinic (Barcelona, Spain) and this study was approved by the Hospital Clinic of Barcelona ethics committee (2012/7635). Clinical parameters are summarized in Supplementary Table III (left column, BCLC). In brief, 50 of the 84 received sorafenib after surgery or TACE treatment, while 34 received it as the first treatment. The median duration of the treatment was 6.15 months (IQR 2.94–10.64) and the median follow-up of patients was 11.13 months (IQR 6.89–20.04).

### Statistical analysis

Laboratory data are represented as mean ± SEM. Data are represented as fold change referred to non-treated control conditions. Statistical differences were measured using an unpaired two-sided Student *t*-test or the Welch’s test whenever unequal variances were found or paired *t*-test as indicated. Clinical data are represented as a median and interquartile range [IQR 25th–75th percentiles] for continuous or ordinal variables, and absolute frequency and percentages (%) for categorical. Nonparametric tests (*U* Mann–Whitney test or Wilcoxon signed-rank test for paired analysis) were used for continuous or ordinal variables, except in cases where the data shows a normal distribution, specifically evaluated in each case by histograms. Differences between categorical variables were assessed by Pearson Chi-square, or Fisher exact test when appropriate. Time to event variables was described using the Kaplan–Meier method and the survival functions were compared with the log-rank test. Hazard ratios (HR) and their 95% confidence intervals (95%) were estimated with survival Cox models. For statistically significant Cox models, the sensitivity, specificity, area under the curve (AUC), and their 95%CI were estimated in relevant time-points. Also, Harrell’s c-statistic concordance and their 95%CI were estimated.

Statistical analyses on Newcastle patients were performed with SPSS, version 21 (SPSS Inc. Chicago, USA) licensed to Newcastle University, and on BCLC patients, the statistical analyses were performed with SAS 9.4 software (SAS Institute, Cary, NC, USA). The level of significance was set at the two-sided 5% level.

### Animals

A xenograft murine model was established by injecting ≈5 × 10^6^ Huh7 hepatoma cells into both flanks of NU(NCr)-Foxn1^nu^ athymic nude female mice (*n* = 5, 4 months old, male). One week after cell injection, tumor masses were monitored and when the tumors reached a volume of ≈200 mm^3^, mice received a daily dose of sorafenib (15 mg/kg body weight, intragastrically) for 3 weeks or less until the tumors reached 2000 mm^3^. MiR control (miR-Ctrl) or miR-518d-5p was overexpressed directly by intratumoral injection (10 µg/tumor, twice a week) at each flank with jetPEI (Polyplus). Mice were euthanized at the end of the treatment or when the tumor exceeded 2000 mm^3^. Tumor volume was calculated according to the formula: volume = (*a* × *b*^2^)/2, where “*a*” is the largest diameter and “*b*” is the perpendicular diameter. At sacrifice, tumors were collected for both molecular biology and histopathology. CIC bioGUNE’s Animal Care and Use Committee and the competent authority (Diputación de Bizkaia) approved the animal procedures.

### Studies in HCC cell lines

In vitro experiments were performed using the human hepatoma BCLC3 cell line, obtained and characterized as previously reported^37^. The THLE2, Huh7, PLC, and HepG2 cell lines were purchased from ATCC. To carry out in vitro miR-518d-5p inhibition and overexpression, human hepatoma BCLC3 and Huh7 cells were transfected with miRIDIAN microRNA Hairpin Inhibitor/Mimic miR-518d-5p or an unrelated miR-Ctrl using DharmaFECT transfection reagent (Dharmacon, USA) at 25 nM in the culture medium following the manufacturer’s procedure. The study of the response to sorafenib treatment in hepatoma cell lines was carried out using BCLC3 and Huh7 hepatoma cells that were cultured with 10 µM of sorafenib (Selleckchem, USA) for 24 h in a 10% FBS culture medium. Cellular assays were performed at least three times independently. A minimum of three replicates was included per experiment

### Luciferase reporter assays

Human c-Jun 3′UTR clone in pMirTarget (cat. number SC213791) and empty vector pMiRTarget (cat. number PS100062) were purchased from OriGene (USA). BCLC3 and Huh7 cells were transfected with the pMir or pMir-c-Jun-3′UTR vectors together with miRIDIAN microRNA Hairpin Inhibitor/Mimic miR-518d-5p or scrambled siRNA using the DharmaFECT Duo Transfection Reagent (Dharmacon), respectively. Firefly luciferase activity was determined in cell lysates with the Dual-GLO luciferase assay system (Promega, USA).

### Studies with mitochondria: ROS, membrane potential, and colocalization

Mitochondrial reactive oxygen species (ROS) production was determined by MitoSOX Red mitochondrial superoxide indicator (Invitrogen, USA). Cultured cells were loaded with 2 μM MitoSOX Red (10 min, 37 °C in a CO_2_ incubator). Cells were then washed with PBS and fluorescence was read at 510/595 nm (excitation/emission) using a SpectramaxM2 (Molecular Devices, USA). Mitochondrial membrane potential was determined using Tetramethylrhodamine (TMRM) indicator (Invitrogen). Cultured cells were collected and incubated in PBS with TMRM 0.5 µM (30 min, 37 °C in a CO_2_ incubator). Fluorescence was read at 548/574 nm (excitation/emission) using a SpectramaxM2 (Molecular Devices). Total mitochondria were determined by MitoTracker^®^ Green FM (Invitrogen) staining following the manufacturer’s recommendations. Cultured cells were loaded with 100 nM MitoTracker^®^ Green FM (15 min, 37 °C in a CO_2_ incubator). Cells were washed and directly analyzed at the microscope or fixed with 4% paraformaldehyde for subsequent mitochondrial-PUMA colocalization studies. PUMA staining was performed at 1:200; overnight; 4 C (Cell Signaling 4976) and developed with secondary Rabbit-Cy3 (1:500; 2 h; RT). Pictures were taken with an Axio Imager D1 Upright Fluorescence Microscope (Carl Zeiss AG, Jena, Germany). Quantification of PUMA-MitoTracker^®^ Green co-staining was performed using the Frida Software (FRamework for Image Dataset Analysis) http://bui3.win.ad.jhu.edu/frida/. Change in % area respect untreated control is represented in the figures.

### Real-time respiration studies using Seahorse analyzer technologies

The cellular metabolic profile was determined using a Seahorse XFe24 Analyzer (Seahorse Biosciences, USA), providing real-time measurements of the oxygen consumption rate (OCR) as previously described^27^. In total, 7–8 × 10^3^ cells were plated and transfected in an XF24 cell culture microplate (Seahorse Bioscience) for 16 h (overnight). Then, media was changed and, the next day, Seahorse analysis was performed. Real-time respiration assays were performed using Agilent seahorse DMEM, without bicarbonate and supplemented with, glutamine (1 mM), glucose (10 mM), sodium pyruvate (2 mM). Different assays with or without sorafenib (10 µM) were done. All the pharmacologic inhibitors were administered through cartridges with injector ports on the XF sensor, thus, modulating respiration directly into the cell well during the assay. After analysis, cell density was evaluated by crystal violet, and this value was used to normalize readings.

For the XF Cell Mito Stress Test Kit, used as described in the User Guide (Kit 103015-100, Agilent), the following pharmacologic inhibitors were used: oligomycin (1 μM), for the ATP-coupled oxygen consumption; carbonyl cyanide 4-trifluoromethoxyphenylhydrazone (FCCP) (300 nM), an uncoupling agent that allows maximum electron transport, showing the maximal OXPHOS respiration capacity; and Rotenone (1 µM)/Antimycin (1 µM) mix as a mitochondrial complex I and III inhibitors, respectively.

For the ATP production rate, the assay was assessed simultaneously from glycolysis and mitochondria using label-free technology XF Real-Time ATP Rate Assay kit as described in the User Guide (Kit 103592-100, Agilent). The following pharmacologic inhibitors were used: oligomycin (1 µM) and Rotenone (1 µM)/Antimycin (1 µM) mix.

### Cell death and viability analyses

Cell death/viability was determined by two different methods. Cell death was determined by AnnexinV analysis using flow cytometry. Briefly, cells were collected and pelleted for subsequent staining in 100 µl of AnnexinV Buffer with 2 µl of propidium iodide (PI) and 2 µl of AnnexinV (FITC) for 15 min, at RT, protected from light (BD Biosciences, USA). Stained cells were analyzed in a FACS Canto Cytometer (BD Biosciences).

For viability analyses, cells were stained and fixed with 0.1% Crystal violet in 20% methanol solution at RT for 40 min. Then cells were washed and dried and incubated in 10% acetic acid for 1 h at RT and finally measured using a Spectramax M3 spectrophotometer.

### RNA isolation and quantitative real-time PCR

RNA was isolated with Trizol (Invitrogen), and its concentration and integrity were determined. PCRs were performed using iQ™ SYBR® Green Supermix (Biorad) and the Bio-Rad iCycler thermocycler (Bio-Rad, Hercules, CA). The Ct values were extrapolated to a standard curve, and data was then normalized to the housekeeping expression (ARP).

### Protein isolation and western blotting

Extraction of total protein from cultured cells and livers was performed as described^38^. In total, 4–25 µg of protein were electrophoresed on sodium dodecyl sulfatepolyacrylamide gels and transferred onto membranes. Band intensities were quantified using the ImageJ software and normalized to the β-ACTIN housekeeping.

### Immunohistochemistry

Paraffin-embedded liver samples were sectioned, dewaxed, and hydrated. Immunohistochemistry was performed as previously described^39^.

### TUNEL immunofluorescence

Tumor paraffin-embedded tissue sections were first dewaxed and hydrated, then were stained using in situ cell death detection kit (Roche). Briefly, samples were incubated 3% H_2_O_2_ (diluted in MeOH), second, 20 min of incubation with Proteinase K to unmask or display the epitopes. Finally, we incubate the enzyme 2 h at 37°, samples were mounted with DAPI. Ten to twenty random images per sample were taken with a ×20 objective in Upright Fluorescent Microscope (Axioimager D1). The stained area percentage of each sample was calculated using FIJI (ImageJ) https://imagej.net/Fiji.

### MiRNA quantitative real-time PCR

RT-PCR was performed for miR-518d-5p following a TaqMan® MicroRNA Reverse Transcription Kit (Life Technologies, USA) procedure using 10–50 ng of total RNA. qPCR was performed with the TaqMan Universal PCR Master Mix No AmpErase UNG kit following the manufacturer’s procedure. miR-518d-5p expression levels were normalized with the U6 snRNA.

### MiRNA isolation and quantification in serum

MiRNA was isolated from serum with the miRNeasy Serum/Plasma Kit (Qiagen, Germany) following the manufacturer’s procedure. RT/q-PCR was performed as described above. miR-518d-5p levels were normalized with the miR-39 Spike-In Control for the Newcastle and Barcelona BCLC cohorts and with Spike-In sp4 for healthy and HCC patients from the Donostia University Hospital.

### Supplemental material

Detailed material of the study is provided in the Supplemental material document.

## Results

### miR-518d-5p is overexpressed in human hepatoma cells

**Fig. 1:**
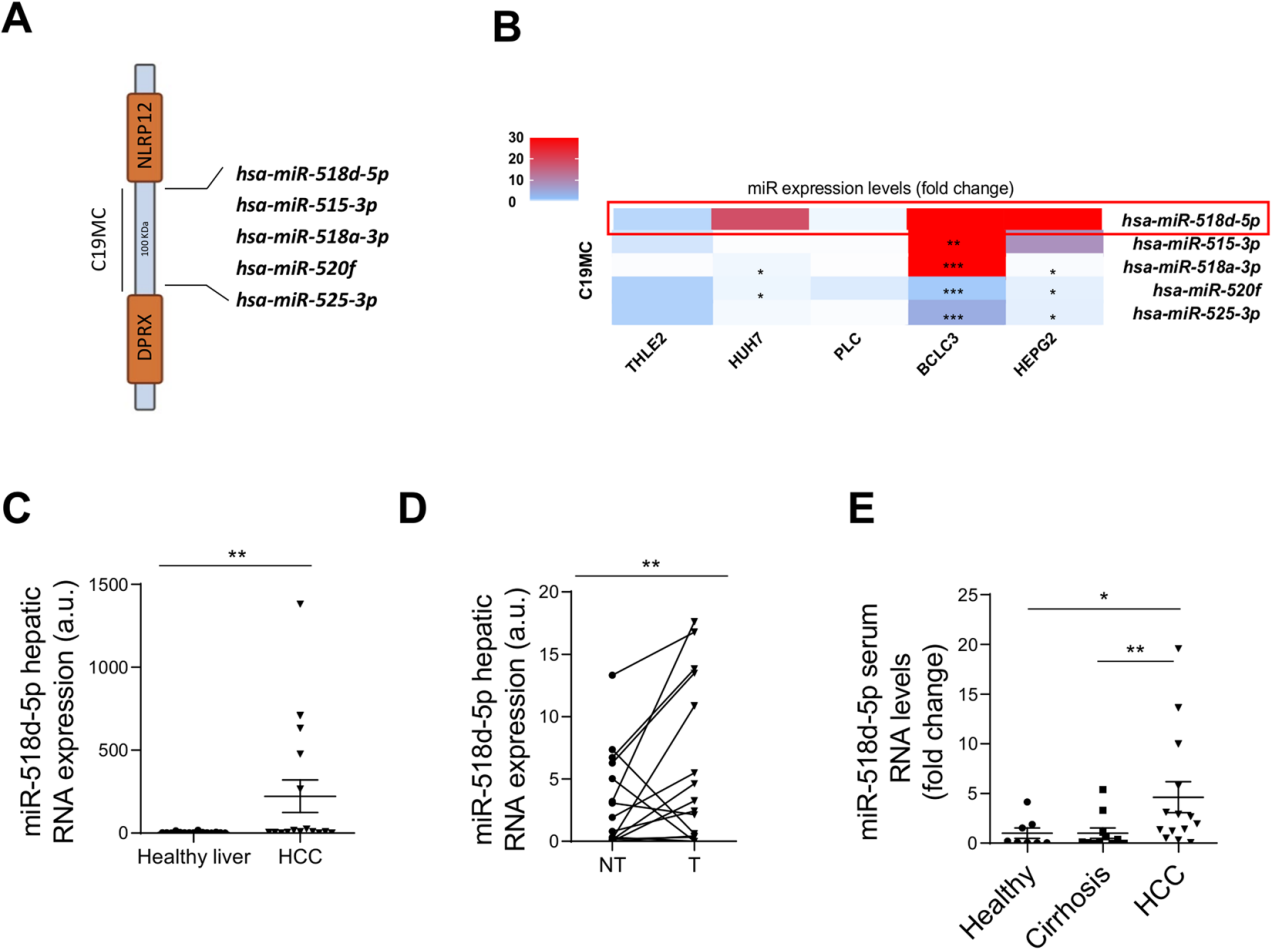
Hepatic and circulating miR-518d-5p levels in hepatocellular carcinoma patients. **A** Schematic representation of the C19MC microRNA cluster, highlighting the five miRNAS analyzed. **B** Heat map depiction of the levels of the five miRNAs analyzed in the indicated cell lines. **C** MiR-518d-5p RNA expression in liver biopsies from a cohort of healthy and HCC patients (Mann–Whitney test), and in a second cohort (**D**) of HCC patients of non-tumoral tissue (NT) and tumoral tissue (T) liver biopsies (Wilcoxon signed-rank test). **E** Serum circulating levels of miR-518d-5p in healthy cirrhotic and HCC patients. Data presented as mean ± SEM. *p < 0.05; **p < 0.01.

The relevance of microRNAs in diagnosis and prognosis in HCC is rapidly growing. Notably, the largest human miRNA cluster, C19MC, has been reported to be altered in HCC patients with protumorogenic and metastatic activity^34,40^. Consequently, we selected five top up-regulated miRNAs from the C19MC described to be involved in HCC^40^. Selected miRNAs were miR-518d–5p, miR-515-3p, miR-518a-3p, miR-520f, miR-525-3p (Fig. 1A). To screen for the potential value of these microRNAs as therapeutic targets in HCC, we first analyzed their expression levels in different hepatoma cell lines compared to the immortalized liver human control cell line, THLE-2. MiR-518d-5p had the most consistent upregulation between the different hepatoma cell lines while others were not consistently altered or were even decreased (Fig. 1B). With this premise, we focused on miR-518d-5p to further study its role in HCC. Thus, we evaluated miR-518d-5p levels in tissues from 36 patients with HCC. These included a cohort of 16 patients with HCC of diverse etiology, in which miR-518d-5p was overexpressed compared with healthy subjects (Fig. 1C) (Supplementary Table I). Similarly, in an independent validation cohort of 20 HCC patients^35^ (baseline-paired HCC and surrounding tissue), increased miR-518d-5p in liver tumors was confirmed (Fig. 1D).

### Increased serum miR-518d-5p levels are associated with HCC

Finding new biomarkers to attend to the needs associated with personalized treatments is a challenge in the oncology field. To examine whether miR-518d-5p is a feasible circulating marker, we analyzed serum miRNA levels from healthy, cirrhotic, and HCC patients. Increased circulating miR-518d-5p levels were detected in the 12 HCC patients whose hepatic levels had been evaluated previously (Fig. 1C), as compared to 10 healthy individuals and 15 cirrhotic patients (Fig. 1E). Thus, increased miR-518d-5p serum levels may have value as an HCC biomarker.

### miR-518d-5p regulates mitochondrial functionality in human hepatoma cells

**Fig. 2:**
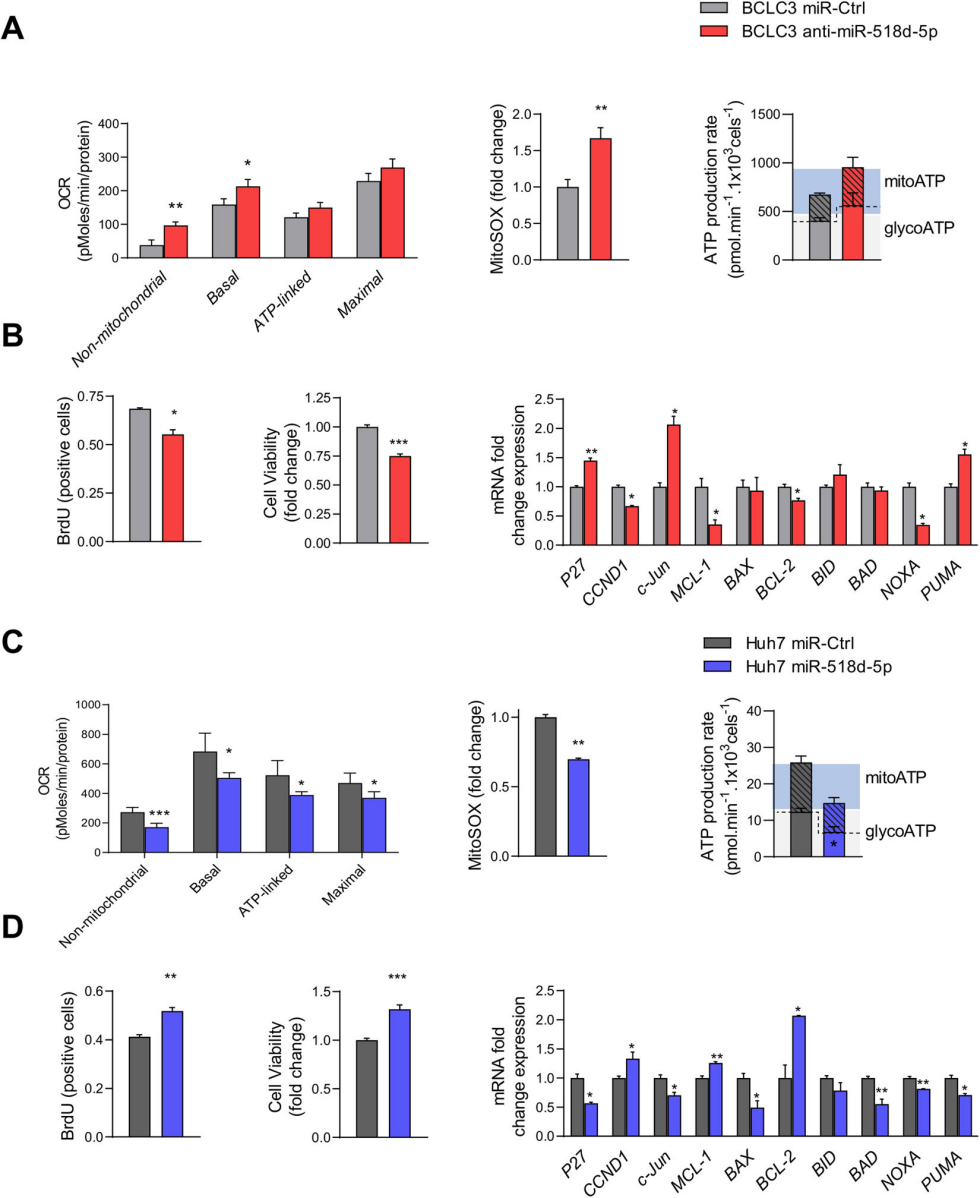
Experimental manipulation of miR-518d-5p expression affects the oncogenic capacity of human hepatoma cell lines. Anti/mimic-miR-518d-5p (25 nM, 48 h) effect in BCLC3 and Huh7 hepatoma cells. **A** Determination of oxygen consumption rate (OCR), mitochondrial ROS (MitoSOX) production, and dual ATP production (glycolysis and mitochondria) levels in BCLC3 cells under miR-518d-5p inhibition. **B** Determination of proliferation (BrdU), survival (Crystal violet), and gene expression (qPCR) of indicated genes in BCLC3 under miR-518d-5p inhibition. **C** Determination of oxygen consumption rate (OCR), mitochondrial ROS (MitoSOX) production, and dual ATP production (glycolysis and mitochondria) levels in Huh7 cells under miR-518d-5p overexpression. **D** Determination of proliferation (BrdU), survival (Crystal violet), and gene expression (qPCR) of indicated genes in Huh7 cells under miR-518d-5p overexpression. Data normalized as fold change vs. control where indicated. Data presented as mean ± SEM. *p* < 0.05; *p < 0.01; **p < 0.001.

Cancer cells are more sensitive to the rapid increase in ROS than normal cells^41^. Therefore, abnormal production of ROS could trigger cell cycle arrest and apoptosis, with the direct participation of mitochondria^42–44^. Results in Fig. 2A indicate that in human hepatoma BCLC3 cells transfected with anti-miR-518d-5p (Supplementary Fig. 1A), mitochondrial ROS levels were elevated (Fig. 2A). The increase in ROS production is in accordance with a higher respiratory capacity after anti-miR-518d-5p administration (Fig. 2A). The quantification of ATP production rate from glycolysis and mitochondria using label-free technology revealed no significant changes in BCLC3 control versus anti-miR-518d-5p cells, albeit a tendency for increased ATP production is detected (Fig. 2A). Moreover, blocking miR-518d-5p induced JNK activity and increased levels of c-Jun at the mRNA and protein levels (Fig. 2B, Supplementary Fig. 1B). As a consequence, proliferation, anti-apoptotic markers, and survival were reduced whilst genes and proteins related to apoptosis were induced (Fig. 2B and Supplementary Fig. 1B).

Next, we determined whether increasing miR-518d-5p levels induced relevant changes in hepatoma cell behavior. To address this, a mimic-miR-518d-5p was transfected into Huh7 human hepatoma cells, where miR-518d-5p expression is much lower than in the BCLC3 cells (Fig. 1B and Supplementary Fig. 1C). Mitochondrial analysis revealed decreased respiratory capacity, ROS production, and lower ATP production from glycolysis and mitochondria (Fig. 2C). MiR-518d-5p-overexpressing cells showed decreased JNK activation, accompanied by increased BrdU and cell viability. Downregulation of c-Jun and other genes related to cell cycle inhibition and apoptosis were also identified in the presence of miR-518d-5p in Huh7 cells. These data further support that this miRNA participates in the regulation of these processes in HCC (Fig. 2D, Supplementary Fig. 1D).

### miR-518d-5p desensitizes liver tumor to sorafenib induced cell death through mitochondrial activity

**Fig. 3:**
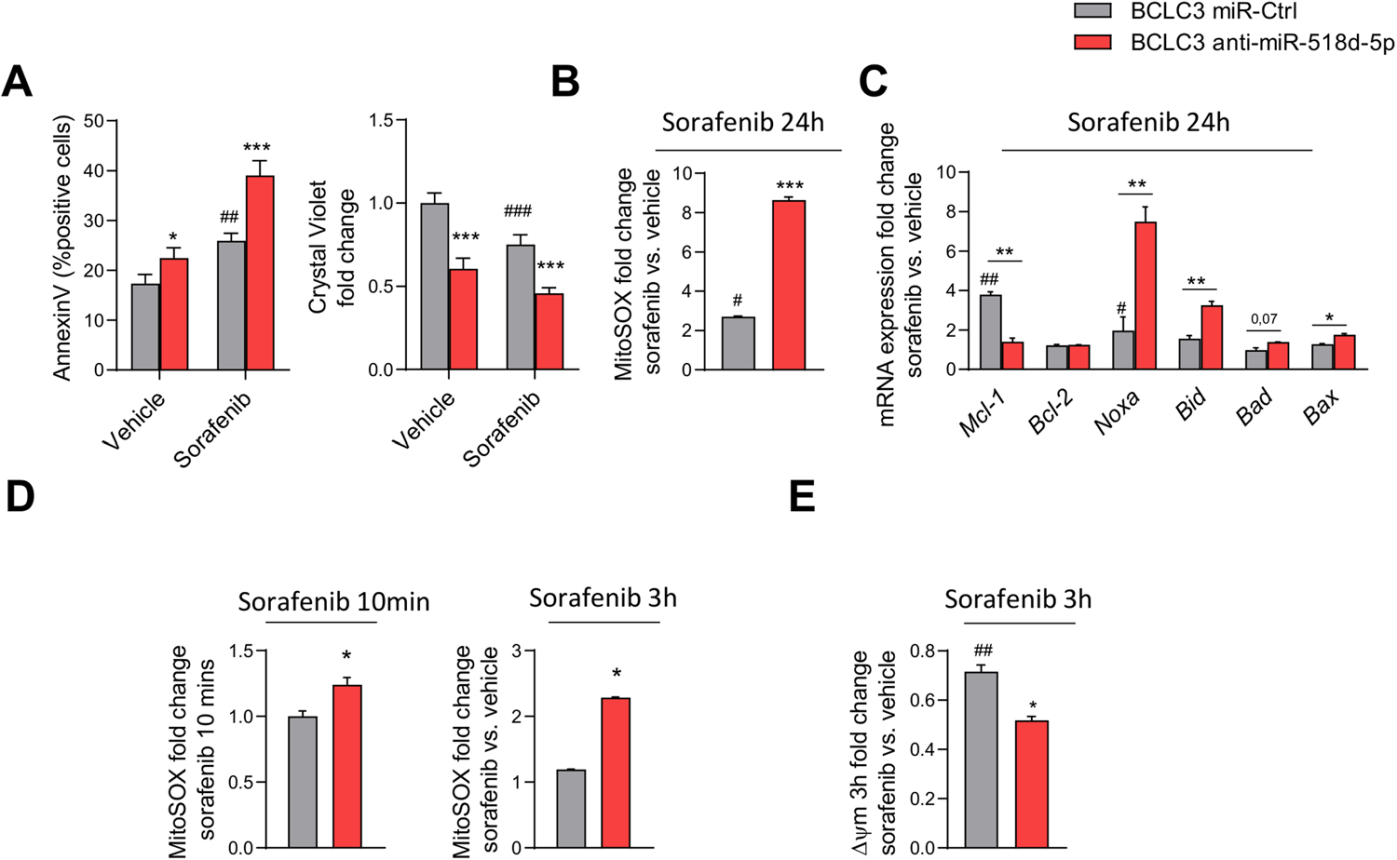
Inhibition of miR-518d-5p enhances sorafenib-induced cell death and mitochondrial dysfunction in human hepatoma cells. **A** Cell viability analysis after sorafenib treatment (24 h, 10 µM) measured by AnnexinV (left) and crystal violet (right). Characterization of sorafenib effect (24 h, 10 µM) by (**B**) mitochondrial ROS production (mitoSOX) and (**C**) gene expression analysis. Determination of mitochondrial function by (**D**) ROS production (mitoSOX) and (**E**) mitochondrial membrane potential (TMRE) at indicated short times of sorafenib treatment. *p < 0.05; **p < 0.01; ***p < 0.001. (*miR-Ctrl vs. anti-miR-518d-5p; # compares sorafenib vs. non-treated ctrl cells. Data presented as mean ± SEM).

The role of miRNAs and mitochondria in conferring drug resistance and susceptibility to apoptotic response has been described by several studies^42–44^. To study the role of miR-518d-5p in mitochondrial functionality and its link to sorafenib-induced apoptosis we designed two different strategies combining anti- or mimic- miR-518d-5p with different time points of sorafenib administration. Apoptosis was studied after 24 h of sorafenib exposure, while mitochondrial functionality was analyzed from 5 min to 3 and 24 h of sorafenib administration (Supplementary Fig. 2A). BCLC3 cells with higher miR-518d-5p levels were more resistant to sorafenib treatment than Huh7 cells (Supplementary Fig. 2B). Sorafenib-incubated BCLC3 cells transfected with anti-miR-518d-5p increased (1.4-fold) the proportion of apoptotic cells (AnnexinV/PI) (Fig. 3A). Sorafenib causes apoptosis by inducing mitochondrial stress and ATP depletion in hepatoma cells^45–47^. Notably, combined sorafenib (24 h) plus anti-miR-518d-5p in BCLC3 cells potentiated mitochondrial stress and a fourfold increase in ROS production (MitoSOX) (Fig. 3B). In agreement, different Bcl-2-related anti- and pro-apoptotic genes (*MCL-1, BCL2, NOXA, BID, BAD*, *BAX*, and PUMA) were regulated after anti-miR-518d-5p and sorafenib treatment (Figs. 3C and 5A). To determine the involvement of mitochondrial dysfunction in sorafenib induced cell death in our model, ROS was assessed at shorter times (10 min and 3 h) after sorafenib treatment, a time-point where cell death is still not significant (data not shown), revealing enhanced ROS production in anti-miR-518d-5p hepatoma cells (Fig. 3D). In addition, no differences in mitochondrial transmembrane potential (Δ*Ψ*_*m*_) were observed at 5 and 10 min of sorafenib treatment, while prolonged exposure rendered a reduction on it in the presence of anti-miR-518d-5p in BCLC3 cells (Supplementary Fig. 3A, E). This effect could be produced by an impairment in the respiratory chain. Thus, the anti-miR-518d-5p treatment plus sorafenib was evaluated by seahorse. Although in these circumstances, anti-miR-518d-5p increased OCR that could explain the higher mitochondrial ROS production and the consequent cytotoxic effects, sorafenib treatment produced more pronounced OCR reduction as a readout of impairment in mitochondrial respiration (Supplementary Fig. 3C).

**Fig. 4:**
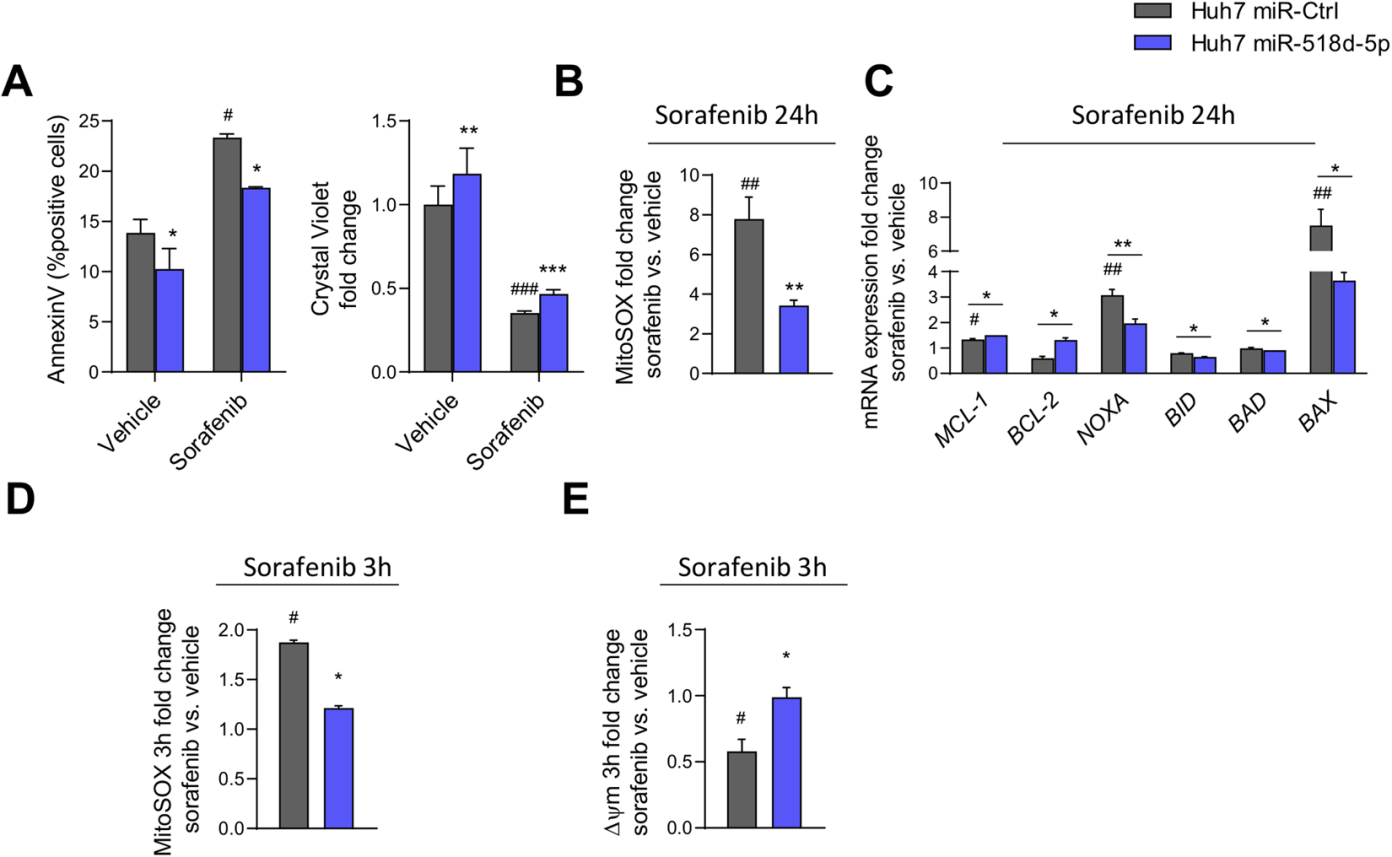
Overexpression of miR-518d-5p induces sorafenib resistance in human hepatoma cells. **A** Cell viability analysis after sorafenib treatment (24 h, 10 µM) measured by AnnexinV (left) and crystal violet (right). Sorafenib effect (24 h, 10 µM) by (**B**) mitochondrial ROS production (mitoSOX) and (**C**) gene expression analysis. Determination of mitochondrial function by analysis of (**D**) ROS production (mitoSOX) and (**E**) mitochondrial membrane potential (TMRE) at 3 h of sorafenib treatment. *p* < 0.05.; *p < 0.01; **p < 0.001. (*miR-Ctrl vs. mimic-miR-518d-5p; # compares sorafenib vs. non-treated ctrl cells. Data are represented as fold change referred to the non-treated control condition. Data presented as mean ± SEM).

On the contrary, experimental overexpression of miR-518d-5p in Huh7 cells reduced apoptosis by FACS-AnnexinV analysis and improved survival upon sorafenib treatment (Fig. 4A). MiR-518d-5p overexpression in Huh7 sorafenib treated cells (24 h) corroborated these data with a reduction close to fourfold in mitochondrial ROS (Fig. 4B). This effect was again accompanied by the regulation of different *Bcl-2*-related genes involved in cell death (Fig. 4C). Moreover, the reduction of mitochondrial ROS, increase of Δ*Ψ*_*m*_ at 3 h, and the reduction of respiratory capacity in hepatoma cells treated with sorafenib plus miR-518d-5p during short time periods (3 h) indicated resistance to sorafenib (Supplementary Figs. 3B, D and 4D, E). Respiration studies showed the opposite effect to sorafenib treatment in miR-518d-5p Huh7 overexpressing cells (Supplementary Fig. 3D), further supporting the role of miR-518d-5p in the regulation of mitochondrial function.

Overall, our results indicate that miR-518d-5p induces sorafenib resistance in hepatoma cells. Mitochondrial activity was lower in the presence of elevated miR-518d-5p levels, associated with reduced ROS production and a chemoresistance phenotype.

### miR-518d-5p targets c-Jun and PUMA in human hepatoma cells

**Fig. 5:**
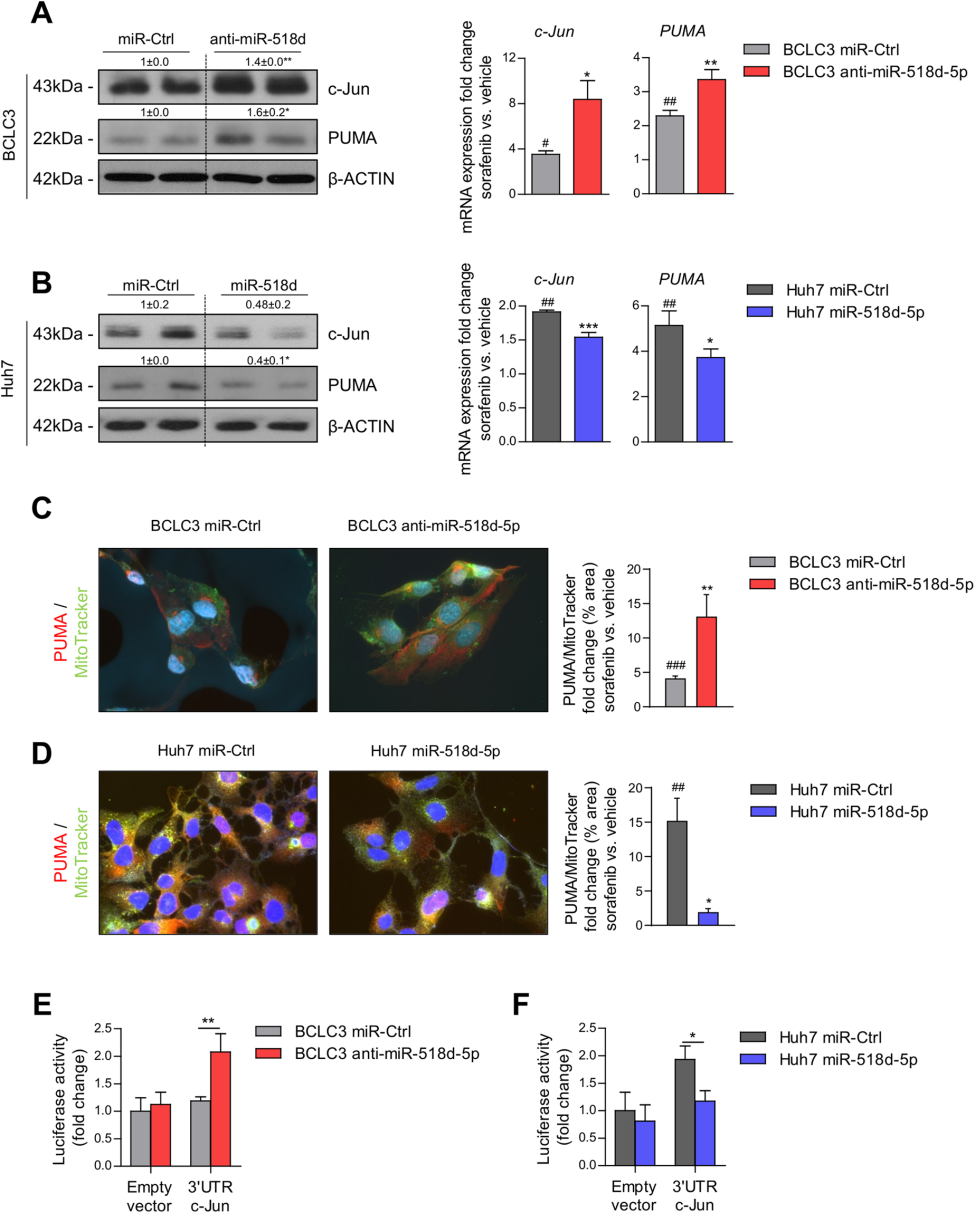
Involvement of miR-518d-5p/c-Jun/PUMA axis in sorafenib-induced cell death in hepatoma cells. WB and qPCR analysis of c-Jun and PUMA in (**A**) BCLC3 and (**B**) Huh7 hepatoma cells under sorafenib treatment (24 h, 10 µM) and miR-518d inhibition/overexpression. Analysis of PUMA mitochondrial subcellular localization by double PUMA/MitoTracker staining under 3 h of sorafenib treatment in BCLC3 (**C**) and (**D**) Huh7 cells during miR-518d-5p inhibition/overexpression. Luciferase reporter assay of c-Jun-3′UTR in (**E**) BCLC3 and (**F**) Huh7 hepatoma cell lines after miR-518d-5p inhibition and overexpression, respectively. Normalized data referred to untreated control in each cell line. *p < 0.05; **p < 0.01; ***p < 0.001. (*miR-Ctrl vs. miR-518d-5p; # compares sorafenib vs. non-treated ctrl cells. Data are represented as fold change referred to the non-treated control condition. Data presented as mean ± SEM).

**Fig. 6:**
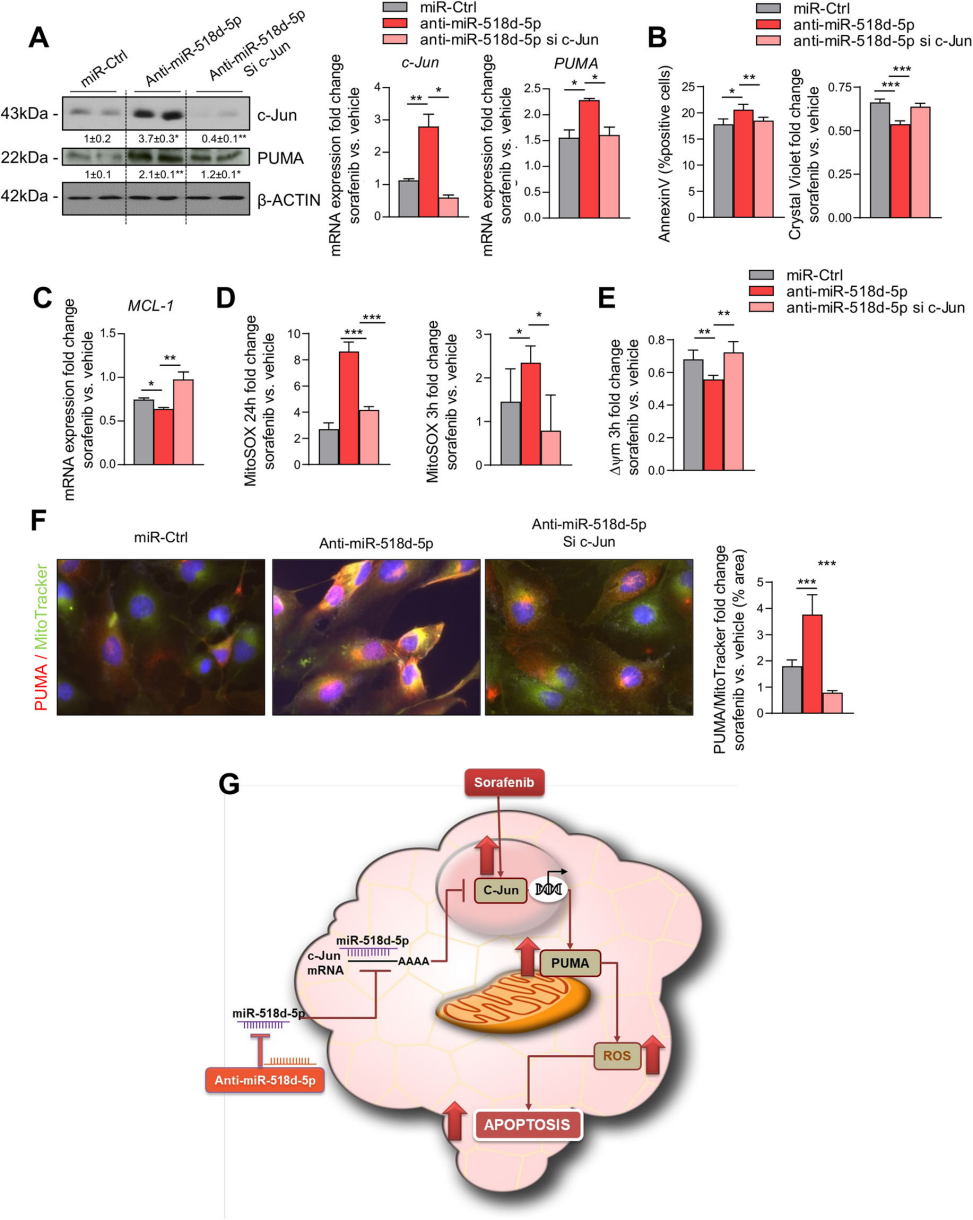
Specific silencing of c-Jun reverts anti-miR-518d-5p proapoptotic effect in BCLC3 cells. **A** WB and qPCR analysis of c-Jun and PUMA in BCLC3 after sorafenib treatment (24 h, 10 µM) and miR-518d-5p inhibition and silencing of c-Jun. **B** Cell death analysis measured by AnnexinV and crystal violet and (**C**) qPCR analysis of indicated survival-related genes in response to miR-518d-5p inhibition and c-Jun silencing in sorafenib treated cells (24 h, 10 µM). Determination of (**D**) mitochondrial ROS production and (**E**) mitochondrial membrane potential after sorafenib treatment at indicated time points. **F** Analysis of PUMA mitochondrial subcellular localization by double PUMA/MitoTracker staining in sorafenib-treated cells (3 h, 10 µM). **G** Summary of the proposed model of miR-518d-5p role in sorafenib resistance in HCC through inhibition of c-Jun-mediated mitochondrial dysfunction and apoptosis. *p < 0.05; **p < 0.01; ***p < 0.001.# compares sorafenib vs. non-treated ctrl cells. Data are represented as fold change referred to the non-treated control condition. Data presented as mean ± SEM.

c-Jun has been previously implicated in sorafenib-induced apoptosis^14–17^. Upregulation of c-Jun and PUMA was the readout of the combinatory anti-miR-518d-5p plus sorafenib in BCLC3 cells (Figs. 5A and 6A), while the overexpression of mimic-miR-518d-5p in the presence of sorafenib resulted in a reduction of both apoptotic markers in Huh7 (Fig. 5B). Indeed, chemoresistant BCLC3 cells showed half of the levels of c-Jun compared to Huh7 cells (Supplementary Fig. 4A, B).

PUMA has been described as the major regulator of apoptotic processes, maintaining mitochondrial outer membrane integrity. PUMA translocation from the cytosol to mitochondria has been shown to promote apoptosis^12^. Moreover, PUMA regulates apoptosis in hepatoma cells incubated with sorafenib^9,16^. Using fluorescence microscopy, we detected that the increase in PUMA observed in BCLC3 cells subjected to anti-miR-518-5p plus sorafenib occurred predominantly in mitochondria (Fig. 5C). The opposite effect was detected in Huh7 cells overexpressing miR-518d-5p under sorafenib, where PUMA levels diminished in mitochondria as showed by co-staining with MitoTracker (Fig. 5D). This suggests a complex regulation of mitochondrial function mediated by miR-518d-5p that may involve cJUN and PUMA.

To clarify the mechanisms underlying miR-518d-5p’s effect on c-Jun and PUMA, an in silico analysis was performed with miRWALK and RNA22 databases to look for predicted miR-518d-5p binding sites in c-Jun or PUMA, identifying a potential target site for miR-518d-5p within the 3′UTR of c-Jun (Supplementary Fig. 5A). To prove a direct interaction between miR-518d-5p and c-Jun, the 3′UTR region of this gene was cloned and the resulting vector (pMir-c-Jun-3′UTR) was employed in a dual-luciferase assay. The cotransfection of the pMiR-c-Jun-3′UTR vector into BCLC3 cells with anti-miR-518d-5p resulted in an increase of the 3′UTR-c-Jun luciferase expression (Fig. 5E). On the contrary, 3′UTR-c-Jun expression in Huh7 cells overexpressing miR-518d-5p decreased luciferase activity (Fig. 5F). Both results confirm miR-518d-5p as a direct repressor of c-Jun in these human hepatoma cell lines. Therefore, targeting miR-518d-5p, which induces the c-Jun-PUMA axis, represents a novel mechanism to induce apoptosis in hepatoma cells and enhance the response to sorafenib.

### c-Jun and PUMA are the effectors of the apoptotic response mediated by miR-518d-5p regulation

To demonstrate that c-Jun targeting is an important mechanism mediating sorafenib resistance by miR-518d-5p in BCLC3 cells, we silenced c-Jun with siRNAs. Cells were transfected with anti-miR-518d-5p and a siRNA for c-Jun and treated with sorafenib. By Immunoblot and qPCR analysis, we observe that silencing c-Jun blocked its increase following anti-miR-518d-5p exposure (Fig. 6A). As expected, c-Jun inhibition decreased mRNA and protein levels of its target PUMA (Fig. 6A). Importantly, c-Jun silencing and PUMA reduction significantly counteracted sorafenib-induced apoptosis in anti-miR-518d-5p treated cells (Fig. 6B). Interestingly, we also observed a regulation in the anti-apoptotic mitochondrial marker MCL-1, reinforcing the regulation of apoptosis mediated by miR-518d/c-Jun in hepatoma cells under sorafenib treatment (Fig. 6C).

We further evaluated the relevance of mitochondria functionality when we silenced c-Jun in combination with anti-miR-518d-5p. Silencing of c-Jun and the described downregulation of PUMA was associated with a reduction of mitochondrial ROS at 24 h and at shorter times (3 h) of sorafenib treatment, even under miR-518d-5p inhibition (Fig. 6D). Reinforcing these results, the loss of mitochondrial transmembrane potential (Δ*Ψ*_*m*_) produced after blocking miR-518d-5p in the presence of sorafenib was avoided when c-Jun and PUMA levels were specifically reduced (Fig. 6E). This indicates that the mitochondrial effect of anti-miR-518d-5p was mainly due to c-Jun-PUMA regulation. Considering the importance of PUMA mitochondrial localization in the apoptotic response mediated by anti-miR-518d-5p, both parameters were evaluated after c-Jun silencing in BCLC3 cells. The PUMA/Mitotracker double staining revealed a reverse effect to that previously observed (Fig. 5C, D) under miR-518d-5p inhibition in sorafenib-treated BCLC3 cells when c-Jun was also inhibited (Fig. 6F). These data demonstrate that miR-518d-5p directly represses c-Jun expression in human hepatoma cells and that c-Jun and its target gene PUMA are specifically involved in miR-518d-5p-mediated sorafenib resistance through the regulation of mitochondrial functionality (Fig. 6G).

Finally, we studied the extent of apoptosis in response to sorafenib due to anti-miR-518d-5p or c-Jun comparing survival rate in cells treated either with anti-miR-518d-5p or overexpressing c-Jun. Our results show that although c-Jun overexpression (Supplementary Fig. 6A) is sufficient to induce sorafenib mediated apoptosis, this effect is not as high as the anti-miR-518d-5p response, suggesting other miR-518d-5p targets may also be involved in sorafenib induced apoptosis (Supplementary Fig. 6B).

### MiR-518d-5p overexpression promotes sorafenib resistance in vivo

**Fig. 7:**
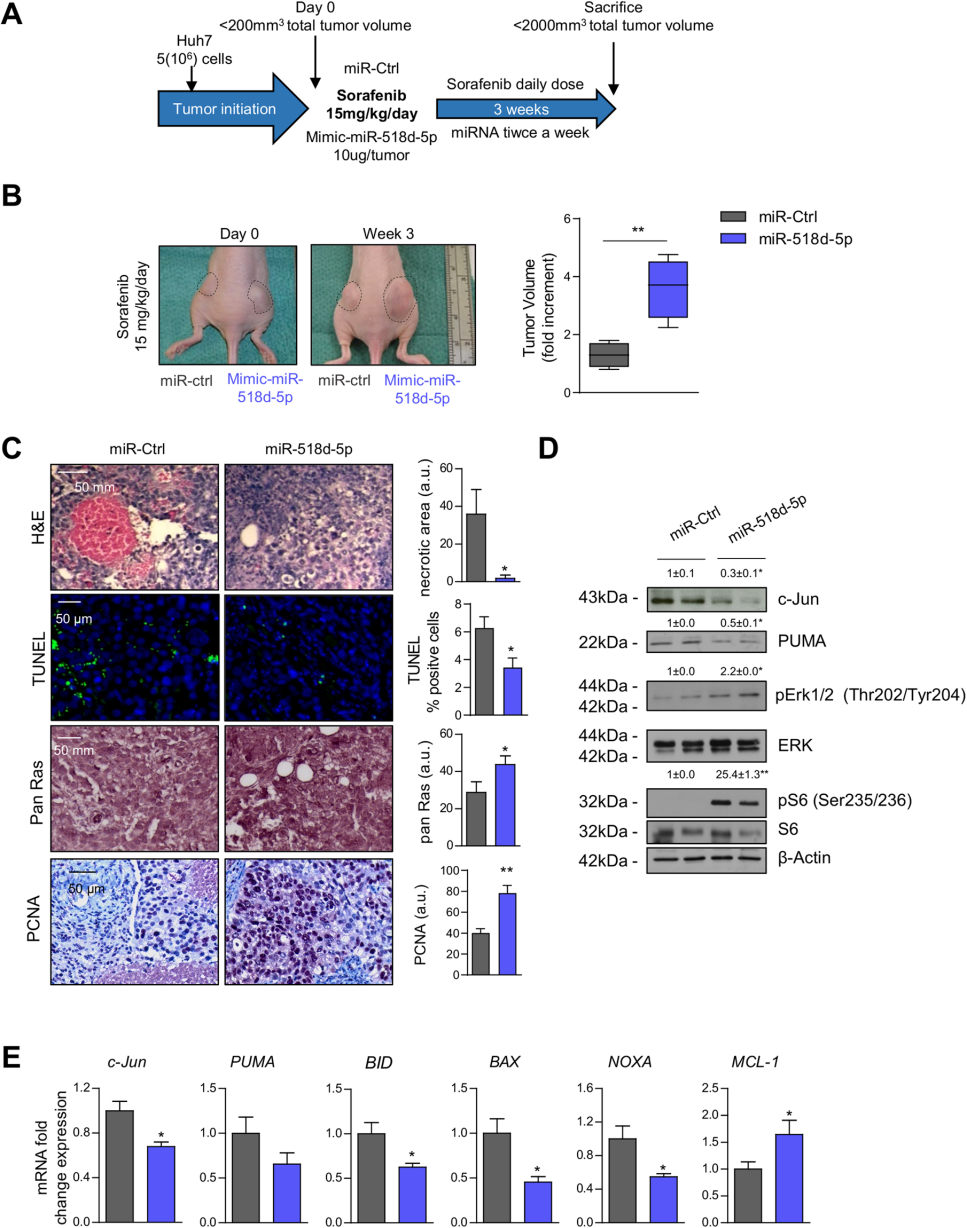
MiR-518d-5p promotes in vivo sorafenib resistance in HCC. **A** Time scheme of xenograft model. **B** Tumor volume fold increment in miR-Ctrl/miR-518d-5p xenograft tumors. **C** H&E, TUNEL, pan-RAS, and PCNA staining in tumor sections of xenografts tumors. **D** WB analysis with indicated antibody and (**E**) mRNA expression analysis of proteins and genes implicated in sorafenib antiproliferative and proapoptotic effect in murine xenografts. *p < 0.05; **p < 0.01. Data are represented as fold change referred to as control. Data presented as mean ± SEM.

To directly confirm the role of miR-518d-5p in sorafenib resistance we employed a xenograft model using Huh7 cells, which are sensitive to sorafenib and express low miR-518d-5p. Tumors were induced at both flanks of the mice and after their formation, miR-Ctrl was induced in one flank tumor, while miR-518d-5p expression was induced in the other, after which mice received a daily dose of sorafenib. Tumor growth and the response to sorafenib were followed for 3 weeks (Fig. 7A). miR-518d-5p overexpression counteracted the effect of sorafenib on tumor growth compared to miR-Control tumors (Fig. 7B). Histological analysis also revealed different effects of sorafenib on proliferation and cell survival/apoptosis, as observed by the decreased necrotic areas and terminal deoxynucleotidyl transferase dUTP nick end labeling (TUNEL) positive cells in the miR-518d-5p overexpressing tumors, together with increased pan-RAS and proliferating cell nuclear antigen (PCNA) (Fig. 7C). These results further suggest a role of miR-518d-5p in resistance to sorafenib-induced apoptosis and growth arrest. Indeed, the activity of ERK and S6 as markers of survival and proliferation were upregulated in miR-518d-5p tumors (Fig. 7D). Remarkably, c-Jun and its targets PUMA, as well as other BCL2 family proteins, were decreased in miR-518d-5p overexpressing tumors (Fig. 7D, E), indicating the anti-apoptotic role of miR-518d-5p.

In summary, the results obtained in this xenograft mouse model provided evidence for an in vivo role of miR-518d-5p in sorafenib drug resistance inferring a modulatory response in mitochondria activity.

### High circulating miR-518d-5p is associated with a poor response to sorafenib treatment

We evaluated miR-518d-5p levels in the sera of 100 patients, including 84 from Barcelona and an independent cohort of 16 patients from Newcastle, using the log value to approximate normality (Supplementary Table III). The underlying etiologies in the Barcelona cohort of patients were mainly viral and alcohol-related, classified as BCLC B and C, while patients from Newcastle predominantly had NAFLD-HCC, classified as BCLC-C. Treatment duration and overall survival time were analyzed based on serum miR-518d-5p levels as a categorical variable, dividing patients into two groups according to the median level of miR-518d-5p (8.84 [0.4–315.05] in all cohort). In the whole combined cohort, patients with miR-518d-5p levels above the median showed a tendency to shorter treatment duration (4.81 [IQR: 2.43–9.8] vs. 7.84 [IQR: 4.57–12.43] months, *p* = 0.07) but not in survival (10.90 (95%CI: 6.54–13.58) vs. 12.39 (95%CI: 10.59–16.17) months, *p* = 0.16) compared to patients with low miR-518d-5p circulating levels (Supplementary Table IV). In the BCLC-C patient’s group, the differences between high and low circulating miR-518d-5p were significantly greater, with a treatment duration of 4.46 [IQR: 1.61–7.76] vs. 8.02 [IQR: 4.57–13.64] months (*p* = 0.006) and survival of 6.49 (95%CI: 4.40–12.75) vs. 12.39 (95%CI: 8.81–17.09) months (*p* = 0.033), with an estimated HR of 1.73 (95%CI: 1.04–2.90, *p*-value = 0.036) (Supplementary Table IV) with a C-statistic of 0.59 (95%CI: 0.52–0.66) (Supplementary Table V). In contrast, no significant differences were found in BCLC-B patients. These data suggest that the circulating levels of miR-518d-5p could be considered as a useful tool to predict sorafenib response in terms of survival in HCC BCLC-C patients.

## Discussion

Changes in miRNA expression are associated with HCC progression^18,20,25,40,48^. The C19MC miRNA cluster includes a set of miRNAs frequently upregulated in HCC and other cancers^34,49^. Nowadays, a clinical challenge in the oncology field, and more specifically in HCC, is the identification of non-invasive biomarkers for prognostic stratification and precision of treatments. Of special relevance is the finding of predictive non-invasive markers of drug resistance to sorafenib, the first-line systemic drug for HCC treatment, and more ambitiously, the quest for innovative approaches that may modulate the response to sorafenib, and thereby their potential use in combinational therapies. In this regard, miRNAs have been linked to the development of resistance to chemotherapeutic agents and, particularly, to sorafenib^28–33,44^. Consistent with other C19MC miRNAs reported previously, our results show increased miR-518d-5p levels in hepatic tumors and serum of HCC patients. Moreover, we provide functional evidence that miR-518d-5p can participate in sorafenib resistance during HCC through enhanced cell proliferation and decreased sorafenib-induced apoptosis. The mechanisms underlying miR-518d-5p-related sorafenib drug resistance were explored.

Different mechanisms of resistance to sorafenib include reduced drug uptake^50^, unresponsiveness to sorafenib by upregulation of *Mcl1*^7^, downregulation of PUMA^9^, and desensitization to TGFβ^16^. PUMA has been proposed as the main mediator of sorafenib-induced apoptosis and can be regulated by different mechanisms including NF-κB and c-Jun^9,10,16^. Indeed, PUMA mitochondrial localization triggers an apoptotic response modulating mitochondrial membrane potential^12^. Our results showed a correlation between PUMA levels, mitochondrial respiration, functionality, ROS production, and response to sorafenib under anti-miR-518d-5p treatment in BCLC3 cells. Indeed, inhibiting miR-518d-5p in the presence of sorafenib promotes a more pronounced reduction in respiratory activity as a readout of electron transport chain functionality. Thus, mitochondrial activity appears as a predictive marker for the response of tumor cells to sorafenib. These results are in agreement with previous studies linking oxidative phosphorylation with mitochondrial dysfunction and sorafenib resistance in hepatoma cells^51^.

Interestingly, a database analysis revealed c-Jun as a potential target of miR-518d-5p, which was confirmed in vitro. Our results indicate a complex regulatory network in the modulation of sorafenib response in vitro and in preclinical animal models between the miR-518d-5p, c-Jun, and its target PUMA. Of note, silencing c-Jun after miR-518d-5p inhibition abrogated the apoptotic response to sorafenib, coinciding with a decrease in the induction of PUMA. These results are in line with previous studies involving c-Jun in PUMA transactivation and apoptosis in hepatocytes and in hepatoma cells treated with sorafenib^10,16^. Moreover, this reinforces the positive role of the JNK/c-Jun pathway activation by sorafenib and other anticancer drugs in the regulation of apoptosis in HCC^11,14,15,17^. In the context of mitochondrial activity, miR-518d-5p confers a survival advantage to liver cancer cells enhancing their buffer capacity against ROS, maintaining membrane integrity, and avoiding apoptosis.

Our data also suggest that miR-518d-5p levels are a promising non-invasive clinical risk factor of the response of HCC patients to sorafenib. Clinical factors associated with a better outcome to sorafenib include lower tumor burden, lack of portal invasion, lack of extrahepatic disease, as well as HCV-HCC rather than other etiologies^52^. Additional predictive biomarkers based on biological traits would be helpful for all patients, but perhaps more so for the growing number of NAFLD-HCC patients, where the chance of benefit is lower and the drug is less well tolerated due to poorer performance status test (PST) and comorbidities. Our results support a tendency of higher risk in advanced HCC, classified as BCLC-C, although it has a low individual prediction capacity, with a C-statistic of 0.59 (0.52–0.66). Further analysis would be required to confirm this tendency. The significantly shorter treatment duration and survival in NAFLD-HCC patients versus other etiologies may all be stage-associated, but it is also known that mitochondrial dysfunction is one of the main mechanisms associated with NAFLD^53^. Hence, it is possible that the impact of miR-518d-5p on mitochondrial activity and ROS varies in the different HCC etiologies under sorafenib treatment.

In summary, our results indicate that sorafenib resistance in liver cancer may be due in part to high miR-518d-5p expression. This is translated into the repression of c-Jun and the inability to efficiently induce transcription PUMA. In turn, decreased PUMA levels lead to membrane potential increase and decreased ROS production that dimmed cancer cell apoptosis by maintaining mitochondrial membrane potential and ROS production at sustained levels. Inhibition of miR-518d-5p loses c-Jun/PUMA repression, triggering the apoptotic process in hepatoma cells. Thus, we propose that strategies aiming at inhibiting miR-518d-5p may contribute to overcoming HCC resistance to sorafenib.

## Supplementary information

Suppl. Fig. 1

Suppl. Fig. 2

Suppl. Fig. 3

Suppl. Fig. 4

Suppl. Fig. 5

Suppl. Fig. 6

Supplemental Figure Legends

Supplemental material
