# Supplementary figures and images for: Anti-miR-518d-5p overcomes liver tumor cell death resistance through mitochondrial activity
Source: Cell Death Dis. 2021 May 28;12(6):555. doi: 10.1038/s41419-021-03827-0 (PMC8163806; doi:10.1038/s41419-021-03827-0)

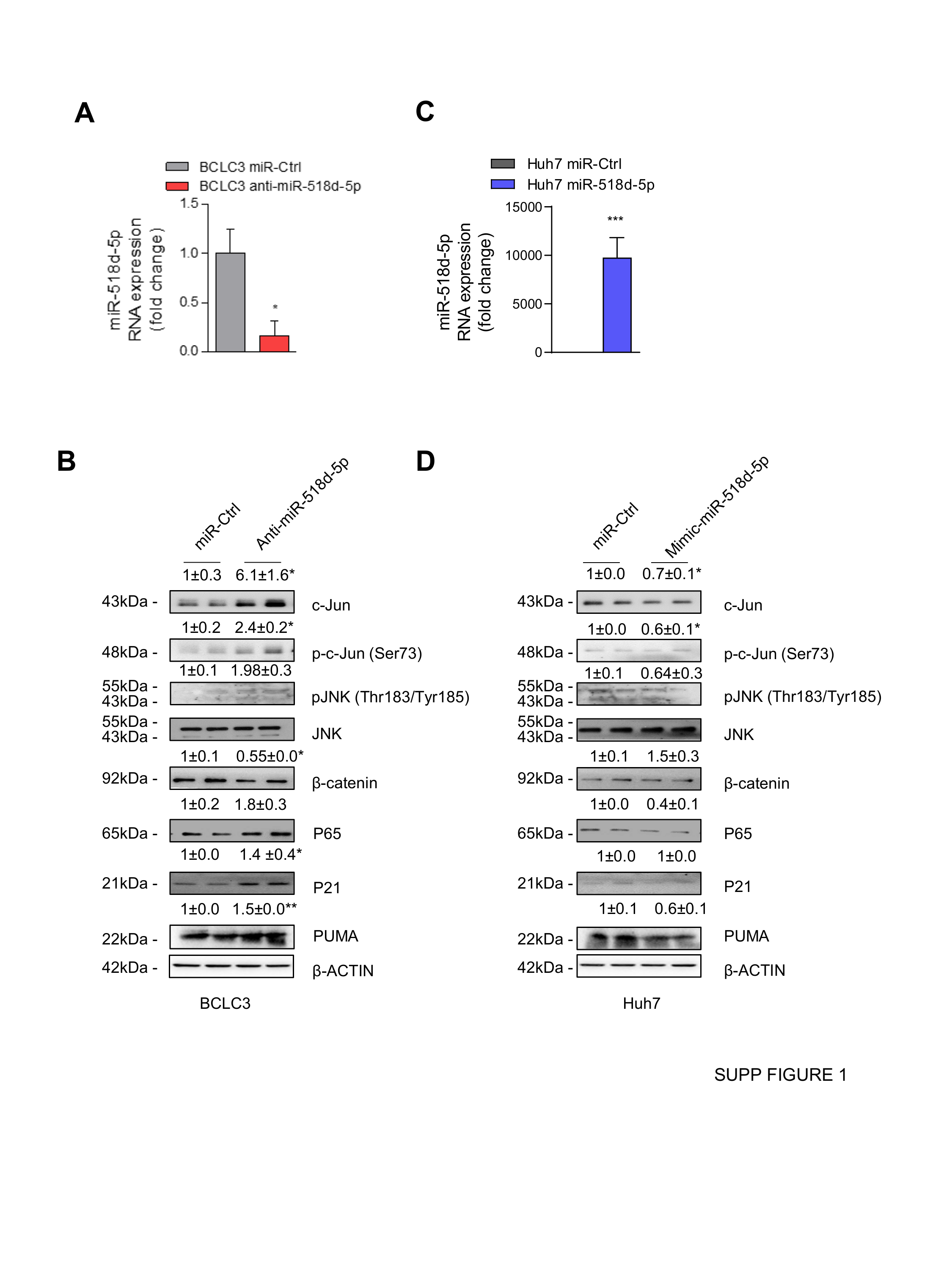

Supplement: Supplementary file 1 — Suppl. Fig. 1 [file 41419_2021_3827_MOESM1_ESM.tif]

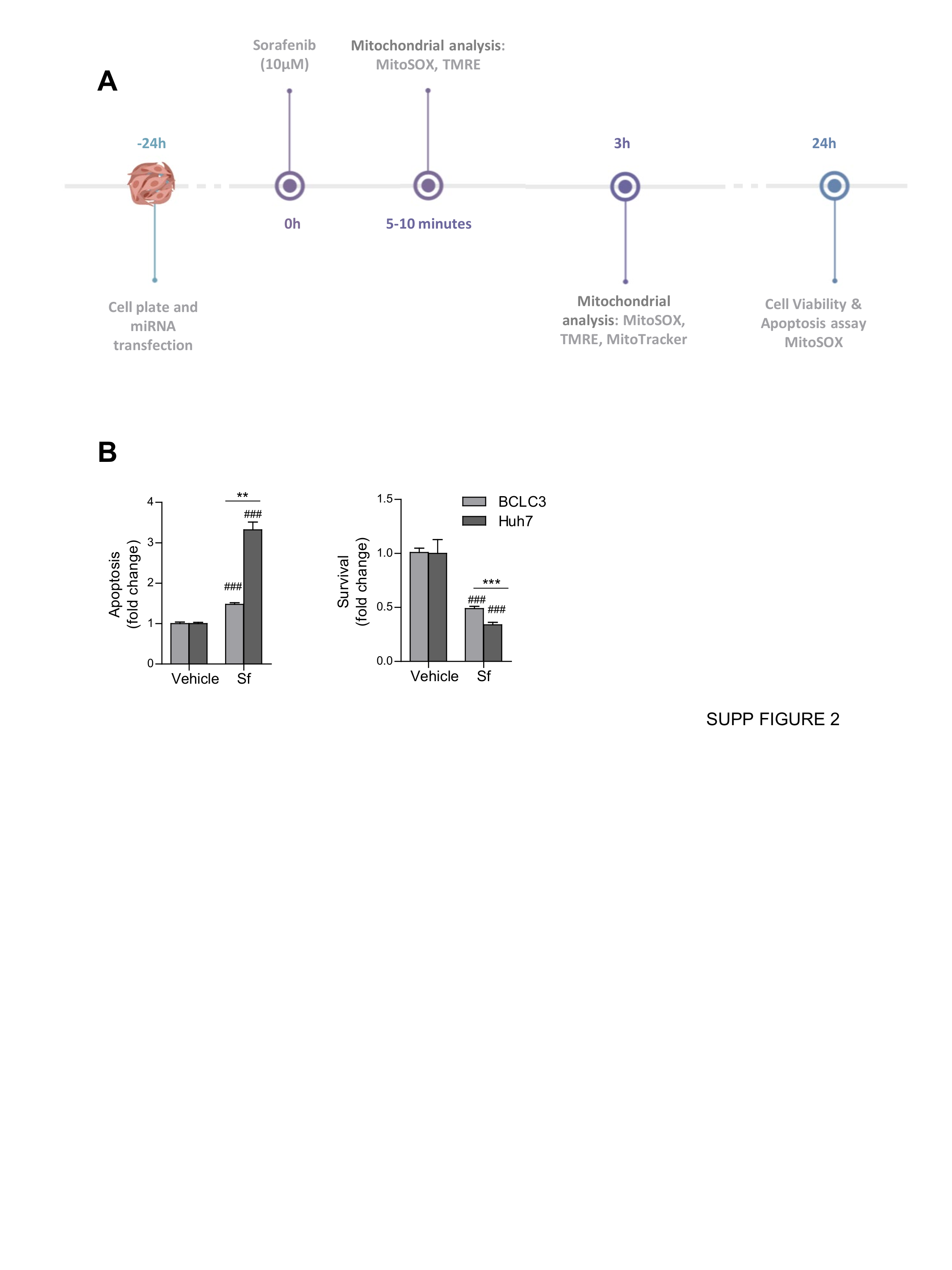

Supplement: Supplementary file 2 — Suppl. Fig. 2 [file 41419_2021_3827_MOESM2_ESM.tif]

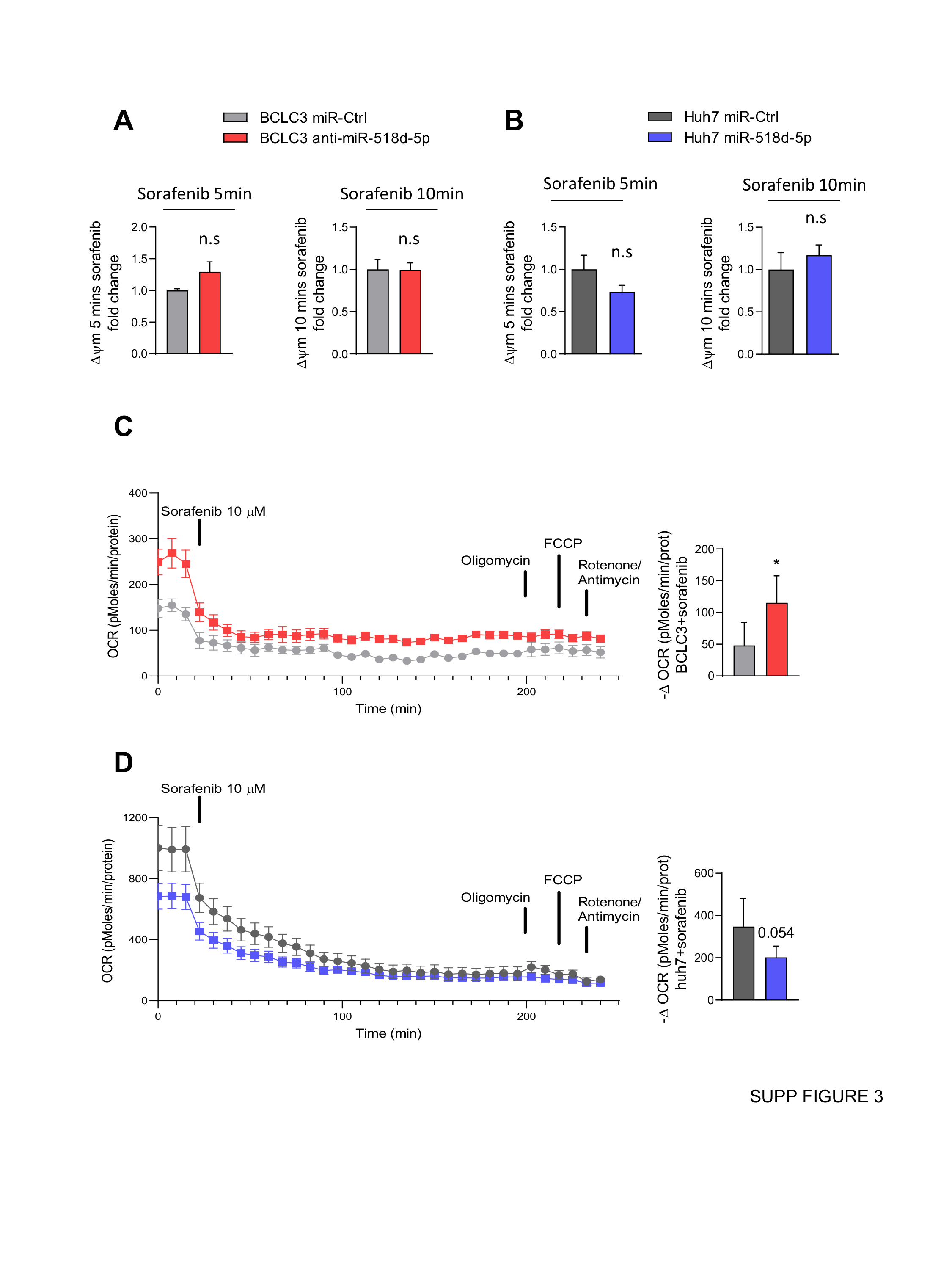

Supplement: Supplementary file 3 — Suppl. Fig. 3 [file 41419_2021_3827_MOESM3_ESM.tif]

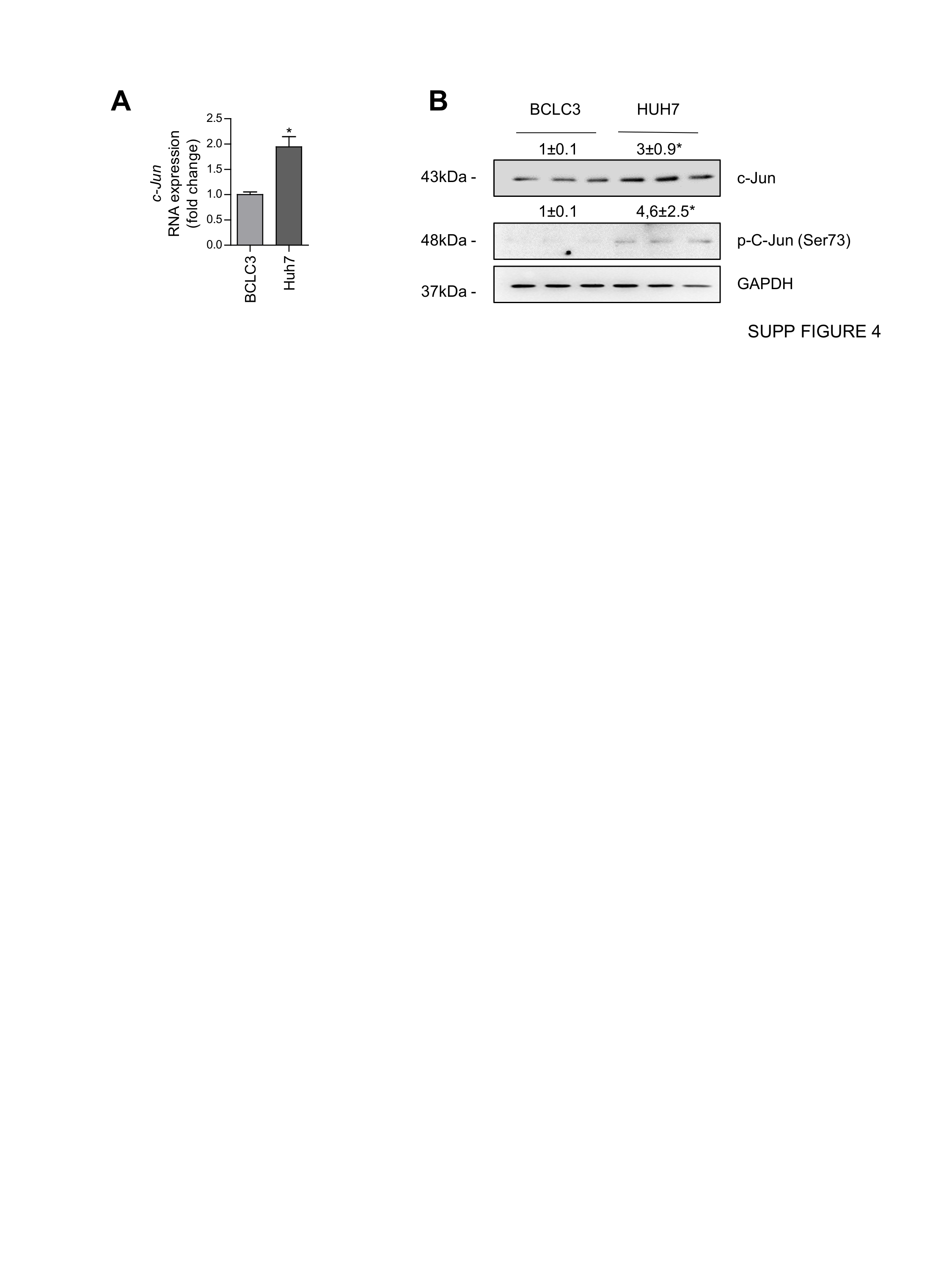

Supplement: Supplementary file 4 — Suppl. Fig. 4 [file 41419_2021_3827_MOESM4_ESM.tif]

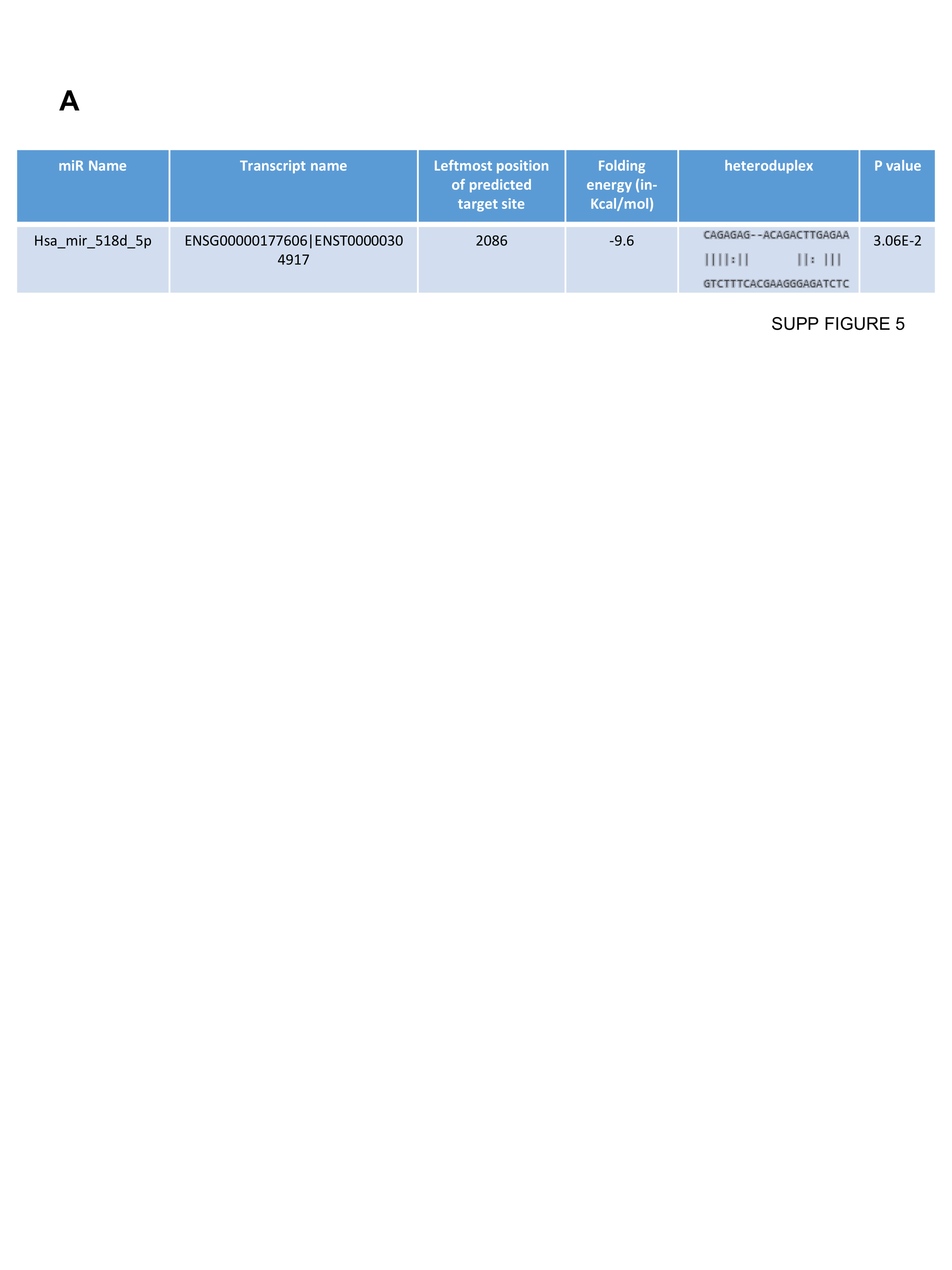

Supplement: Supplementary file 5 — Suppl. Fig. 5 [file 41419_2021_3827_MOESM5_ESM.tif]

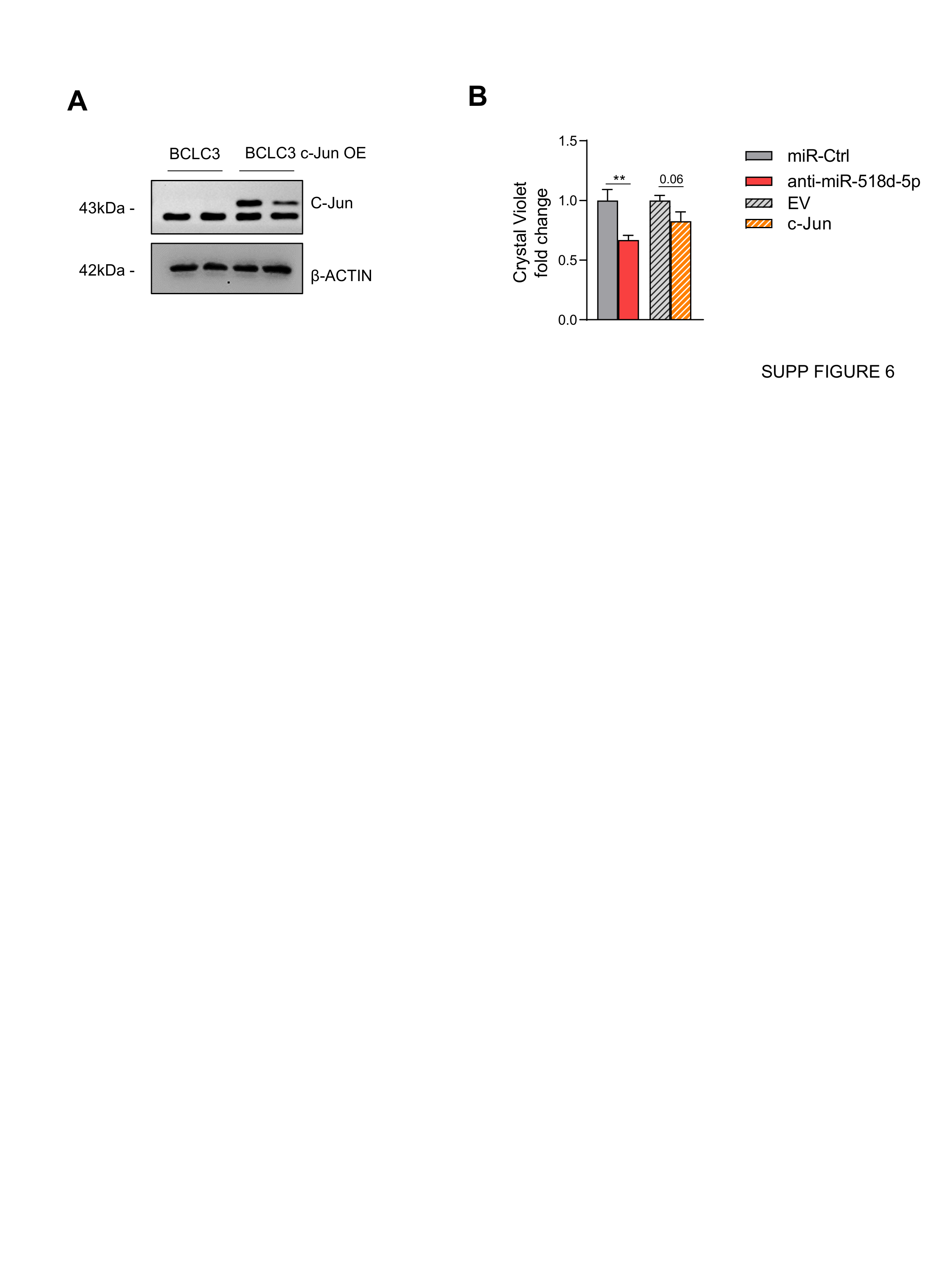

Supplement: Supplementary file 6 — Suppl. Fig. 6 [file 41419_2021_3827_MOESM6_ESM.tif]
