# Supplementary material for: Anti-miR-518d-5p overcomes liver tumor cell death resistance through mitochondrial activity
Source: Cell Death Dis. 2021 May 28;12(6):555. doi: 10.1038/s41419-021-03827-0 (PMC8163806; doi:10.1038/s41419-021-03827-0)
Supplement: Supplementary file 7 — Supplemental Figure Legends [file 41419_2021_3827_MOESM7_ESM.docx]

**Anti-miR-518d-5p overcomes liver tumor cell death resistance through mitochondrial activity**

**Supplemental Figure 1. miR-518d-5p inhibition and overexpression in hepatoma cells.**

(A) RNA levels of miR-518d-5p after 48h transfection of anti-miR-518d-5p (25nM) in BCLC3 cells. (B) WB analysis with indicated antibodies in BCLC3 cells transfected with anti-miR-518d-5p (48h, 25nM). (C) RNA levels of miR-518d-5p in Huh7 overexpressing cells (48h, 25nM). (D) WB analysis with indicated antibodies in Huh7 cells overexpressing miR-518d-5p (48h, 25nM). (E) miR-518d-5p basal levels in BCLC3 and Huh7 hepatoma cells. Data presented as mean ± SEM. p<0.05 *; p<0.01 **; p<0.001.

**Supplemental Figure 2. miR-518d-5p levels affects sorafenib induced cell death in hepatoma cells.**

(A) Time schedule of anti-miR-518d-5p and mimic-miR-518d-5p transfection and sorafenib treatment in BCLC3 and Huh7 hepatoma cells highlighting the main analysis performed at each indicated experimental endpoint. (B) Analysis of cell death (AnnexinV) and survival (crystal violet) in BCLC3 and Huh7 cells under sorafenib (Sf) treatment (24h, 10 µM). Data presented as mean ± SEM. p<0.05 *; p<0.01 **; p<0.001 ***. (*BCLC3 vs. Huh7; # compares vehicle vs. sorafenib treatment in each cell line).

**Supplemental Figure 3. Mitochondrial response to sorafenib after miR-518d-5p modulation in hepatoma cells**

(A) Mitochondrial membrane potential (TMRE) at 5 and 10 minutes of sorafenib treatment in BCLC3 and Huh7 (B) Time dependent effect of sorafenib (10µM) in mitochondrial respiration (OCR) and drug response, followed by graph representation of time 4 vs time 3 in BCLC3. (C) Time dependent effect of sorafenib (10µM) in mitochondrial respiration (OCR) and drug response, followed by graph representation of time 4 vs time 3 in BCLC3 and (D) Huh7 cells. Data presented as mean ± SEM. *; p<0.01 **; p<0.001 (*miR-Ctrl vs. mimic-miR-518d-5p or anti-miR-518d-5p).

**Supplemental Figure ~~4~~. *c-Jun* expression in hepatoma cells.**

(A) mRNA expression measured by qPCR analysis of *c-Jun* in BCLC3 and Huh7 hepatoma cell lines. (B) WB analysis of c-Jun and phospho-c-Jun (Ser73) in BCLC3 and Huh7 hepatoma cell lines. Data presented as mean ± SEM. p<0.05 *.

**Supplemental Figure 5. miR-518d-5p targets c-Jun.**

(A) Predicted targeting of miR-518d-5p within the 3’UTR region of c-Jun. The table shows c-JUN predicted target site by RNA22 database with highest (92%) specificity prediction.

**Supplemental Figure 6. miR-518d-5p has more effects in sorafenib induced cell death than c-Jun overexpression.**

(A) WB analysis showing c-Jun overexpression in BCLC3 cells. (B) Survival rate measured by Crystal Violet in BCLC3 cells under sorafenib (24h,10µM) treatment and anti-miR-518d-5p or c-Jun overexpression. Data presented as mean ± SEM. p<0.01 **.
