# Supplementary material for: Anti-miR-518d-5p overcomes liver tumor cell death resistance through mitochondrial activity
Source: Cell Death Dis. 2021 May 28;12(6):555. doi: 10.1038/s41419-021-03827-0 (PMC8163806; doi:10.1038/s41419-021-03827-0)
Supplement: Supplementary file 8 — Supplemental material [file 41419_2021_3827_MOESM8_ESM.doc]

**Anti-miR-518d-5p overcomes liver tumor cell death resistance through mitochondrial activity**

**Supplemental data:**

- **Supplemental Tables I, II, III, IV, V and VI**

**Supplemental Tables**

**Supplemental Table I. Characteristics of HCC patients and healthy individuals that provided liver biopsies**.

| **Variable** |  | **HCC**  **(n = 16)** | **Healthy liver**  **(n = 16)** |
| --- | --- | --- | --- |
| **Female/Male** |  | 1/15 | 8/8 |
| **Age** | <60  60-70  >70 | 7  2  7 | 2  8  6 |
| **Transaminases** | ALT <40  ALT>40  AST <40  AST>40 | 8  8  7  9 | 14  2  14  2 |
| **Etiology** | VHB+VHD  Cirrhosis VHC  Hemochromatosis  Healthy liver  Alcoholic cirrhosis  Unclassified | 1  5  1  2  4  3 |  |

**Supplemental Table II. Characteristics of HCC patients, cirrhotic and healthy individuals that provided serum samples.**

| **Variable** |  | **HCC**  **(n = 14)** | **Cirrhosis**  **(n = 11)** | **Healthy liver**  **(n = 8)** |
| --- | --- | --- | --- | --- |
| **Female/Male** |  | 1/13 | 1/10 | 4/4 |
| **Age** |  | 65±12.1 | 59±6.9 | 65±6.7 |
| **Transaminases** | ALT  AST | 52±34.7  55±37.3 | 54±44.5  80±51.6 | 22±7.6  14±10.2 |
| **Etiology** | VHB+VHD  Cirrhosis VHC  Hemochromatosis  Healthy liver  Alcoholic cirrhosis  Unclassified | 1  4  1  1  4  3 | 11 |  |

**Supplemental Table III. Characteristics of two different cohorts of HCC patients under sorafenib treatment that provided serum samples.**

|  |  | **BCLC**  **(n = 84)** | **Newcastle**  **(n = 16)** |
| --- | --- | --- | --- |
| **Variable** | **Class** | **median [IQR] and (%) or interval** | **median [IQR] and (%) or interval** |
| **BCLC** | **B** | 37 ( 44.05) | 0 (0.00) |
|  | **C** | 47 ( 55.95) | 16 (100) |
| **Child-Pugh** | **No Cirrhotic** | 3 (3.57) | 5 (31.25) |
|  | **A: 5-6** | 68 (80.95) | 10 (62.5) |
|  | **B: 7-8** | 13 ( 15.48 ) | 1 (6.25) |
| **Ascites** | **No** | 67 ( 79.76 ) | 15 (93.75) |
|  | **Yes** | 17 ( 20.24 ) | 1 (6.25) |
| **Ecephalopathy** | **No** | 84 ( 100 ) | 16 (100 ) |
| **Vascular invasion** | **No** | 53 ( 63.1 ) | 11 (68.75) |
|  | **Yes** | 31 ( 36.9 ) | 5 (31.25) |
| **ECOG PS** | **0** | 72 ( 85.71 ) | 6 (37.5) |
|  | **1** | 12 ( 14.29 ) | 10 (62.5) |
| **Previous treatment** | **Naive** | 34 (40.48) | 7 (43.75) |
|  | **Surgery** | 7 (8.33) | 0 (0.00) |
|  | **Locoregional** | 1 (1.19) | 1 (6.25) |
|  | **Percutaneous** | 12 (14.29) | 0 (0.00) |
|  | **TACE** | 18 (21.43) | 0 (0.00) |
|  | **Others** | 12 (14.29) | 8 (50) |
| **Etiology** | **VHC** | 40 (47.62) | 0 (0.00) |
|  | **VHB** | 2 (2.38) | 0 (0.00) |
|  | **Enol** | 23 (27.38) | 4 (25) |
|  | **VHC-Enol** | 5 (5.95) | 0 (0.00) |
|  | **VHB-Enol** | 2 (2.38) | 0 (0.00) |
|  | **NAFLD** | 0 (0.00) | 10 (62.5) |
|  | **Criptogenic** | 1 (1.19) | 0 (0.00) |
|  | **HIV-VHC** | 5 (5.95) | 0 (0.00) |
|  | **Healty liver** | 3 (3.57) | 2 (12.5) |
|  | **cutaneous porphyria** | 1 (1.19) | 0 (0.00) |
|  | **HIV-enol** | 2 (2.38) | 0 (0.00) |
| **M1** | **No** | 65 ( 77.38 ) | 13 (81.25) |
|  | **Yes** | 19 ( 22.62 ) | 3 (18.75) |
| **Gender** | **Female** | 12 ( 14.29 ) | 2 (12.5) |
|  | **Male** | 72 ( 85.71 ) | 14 (87.5) |
| **Age (Years)** |  | 64.12 [ 54.63 to 70.41 ] | 72.5 [66.5 to 76.5] |
| **Follow-up (months)** |  | 11.13 [ 6.89 to 20.04 ] | 13.02 [9.87 to 19.33] |
| **Treatment time (months)** |  | 6.15 [ 2.94 to 10.64 ] | 6.71 [4.28 to 13.66] |
| **Hemoglobin (g/L)** |  | 13.4 [ 12 to 14.7 ] | 13.3 [11.4 to 14.6] |
| **nbgfdw1º1** |  | 83 [ 68 to 94 ] | 104 [95.83 to 108.33] |
| **Total Bilirubin (mg/dL)** |  | 1.1 [ 0.8 to 1.6 ] | 1.3 [0.8 to 1.8] |
| **Conjugated Bilirubin (mg/dL)** |  | 0.5 [ 0.3 to 0.8 ] | NA |
| **ALT (IU/L)** |  | 66 [ 45 to 107 ] | 42 [28.5 to 66] |
| **AST (IU/L)** |  | 71.5 [ 48 to 106 ] | 55 [45 to 73] |
| **Alkaline Phosphatase (IU/L)** |  | 320 [ 226 to 459 ] | 147 [109 to 174.5] |
| **GGT (IU/L)** |  | 164 [ 85 to 280 ] | 205 [134 to 290] |
| **Albumin (g/L)** |  | 38 [ 36 to 42 ] | 41.4 [39 to 44.5] |
| **Alpha-fetoprotein (ng/dL)** |  | 26 [ 6 to 245 ] | 256 [20.5 to 3486] |
| **miR-518d ( 10^6)** |  | **10.62 [ 0.27 to 384.3 ]** | **4.75 [0.73 to 164.1]** |

**Supplemental Table IV. Evaluation of treatment duration (A) and overall survival (B) in two independent cohorts of HCC patients classified attending to circulating miR-518d-5p levels.**

| **Variable** | **Center** | **Number of patients** | **Groups** | **Time, IQR** | **Wilcoxon (p-value)** |
| --- | --- | --- | --- | --- | --- |
| **Treatment duration** | BCLC | 42 | ≤ 10.62 | 7.63 [4.27 to 10.98] | 0.1781 |
|  |  | 42 | >10.62 | 4.75 [2.43 to 9.8] |  |
|  | Newcastle | 8 | ≤ 4.75 | 13.66 [ 9.17 to 32.51] | **0.0054** |
|  |  | 8 | >4.75 | 4.62 [1.61 to 5.74] |  |
|  | BCLC | 34 | ≤ 4.75 | 7.23 [3.78 to 10.29] | 0.5 |
|  |  | 50 | >4.75 | 5.16 [2.56 to 10.98] |  |
|  | Newcastle | 10 | ≤ 10.62 | 11.35 [4.67 to 17.29] | **0.0262** |
|  |  | 6 | >10.62 | 3.90 [0.46 to 6.44] |  |
|  | All | 50 | ≤ 8.84 | 7.84 [4.57 to 12.43] | 0.0747 |
|  |  | 50 | >8.84 | 4.81 [2.43 to 9.8] |  |
| **Treatment duration by BCLC** | BCLC B | 17 | ≤ 8.84 | 7.66 [4.57 to 9.73] | 0.6919 |
|  |  | 20 | >8.84 | 7.64 [3.68 to 13.38] |  |
|  | BCLC C | 33 | ≤ 8.84 | 8.02 [4.57 to 13.64] | **0.0067** |
|  |  | 30 | >8.84 | 4.46 [1.61 to 7.76] |  |
| **Treatment duration by Etiology** | VHC | 19 | ≤ 8.84 | 6.87[ 4.8 to 9.7 ] | 0.9352 |
|  |  | 21 | >8.84 | 7.59[ 2.56 to 14.86 ] |  |
|  | Enol | 14 | ≤ 8.84 | 10.19[ 3.78 to 13.64 ] | **0.044** |
|  |  | 13 | >8.84 | 4.11[ 2.99 to 5.59 ] |  |
|  | VHC-enol | 2 | ≤ 8.84 | 4.82[ 1.61 to 8.02 ] | 0.7728 |
|  |  | 3 | >8.84 | 3.78[ 0.82 to 7.79 ] |  |
|  | NASH | 5 | ≤ 8.84 | 13.28[ 9.43 to 14.04 ] | **0.0367** |
|  |  | 5 | >8.84 | 2.76[ 0.46 to 6.44 ] |  |
|  | HIV-VHC | 2 | ≤ 8.84 | 4.9[ 4.57 to 5.23 ] | 0.7728 |
|  |  | 3 | >8.84 | 7.92[ 4.37 to 11.93 ] |  |
|  | Healthy Liver | 3 | ≤ 8.84 | 3.98[ 2.89 to 4.57 ] | 0.1489 |
|  |  | 2 | >8.84 | 2.02[ 1.61 to 2.43 ] |  |

**(A)**

**(B)**

| **Variable** | **Center** | **Number of patients** | **Groups** | **OS (95%CI)** | **log-rank (p-value)** | **HR (95%CI)** | **p-value** | | |
| --- | --- | --- | --- | --- | --- | --- | --- | --- | --- |
| **Survival** | BCLC | 42 | ≤ 10.62 | 11.41 (9.89 - 13.87) | 0.3509 | 1.23 (0.79 - 1.91) | 0.3520 | | |
|  |  | 42 | >10.62 | 10.98 (6.54 - 14.6) |  |  |  | | |
|  | Newcastle | 8 | ≤ 4.75 | 19.33 (12.39 - 34.19) | **0.0008** | 9.85 (2.01 - 48.21) | **0.0048** | | |
|  |  | 8 | >4.75 | 9.87 (0.33 - 13.58) |  |  |  | | |
|  | BCLC | 34 | ≤ 4.75 | 11.57 (9.89 - 20.12) | 0.1049 | 1.45 (0.92 - 2.28) | 0.1070 | | |
|  |  | 50 | >4.75 | 10.75 (6.9 - 13.31) |  |  |  | | |
|  | Newcastle | 10 | ≤ 10.62 | 17.65 (9.37 - 28.83) | **0.0010** | 8.14 (1.93 - 34.29) | **0.0042** | | |
|  |  | 6 | >10.62 | 7.38 (00.33 - 13.58) |  |  |  | | |
|  | All | 50 | ≤ 8.84 | 12.39 (10.59 - 16.17) | 0.1586 | 1.33 (0.89 - 1.99) | 0.1603 | | |
|  |  | 50 | >8.84 | 10.90 (6.54 - 13.58) |  |  |  | | |
| **Survival by BCLC** | BCLC B | 17 | ≤ 8.84 | 13.25 (10.59 - 33.43) | 0.8288 | 1.08 (0.55 - 2.10) | 0.8294 | | |
|  |  | 20 | >8.84 | 13.96 (8.36 - 21.93) |  |  |  | | |
|  | BCLC C | 33 | ≤ 8.84 | 12.39 (8.81 - 17.09) | **0.0337** | 1.73 (1.04 - 2.90) | **0.0360** | | |
|  |  | 30 | >8.84 | 6.49 (4.40 - 12.75) |  |  |  | | |
| **Survival by Etiology** | VHC | 19 | ≤ 8.84 | 9.89 (7.03 - 20.12) | 0.9043 |  | |  |  |
|  |  | 21 | >8.84 | 10.91 (6.54 - 19.95) |  |  | |  |  |
|  | Enol | 14 | <≤ 8.84 | 13.54 (10.85 - 20.58) | 0.5434 |  | |  |  |
|  |  | 13 | >8.84 | 12.46 (6.15 - 19) |  |  | |  |  |
|  | VHC-enol | 2 | ≤ 8.84 | 12.49 (8.81 - 16.17) | 0.2769 |  | |  |  |
|  |  | 3 | >8.84 | 4.5 (1.35 - 11.05) |  |  | |  |  |
|  | NASH | 5 | ≤ 8.84 | 17.09 (12.46 - 44.67) | **0.0064** |  | |  |  |
|  |  | 5 | >8.84 | 4.4 (0.33 - 13.58) |  |  | |  |  |
|  | HIV-VHC | 2 | ≤ 8.84 | 7.65 (5.33 - 9.96) | 0.3636 |  | |  |  |
|  |  | 3 | >8.84 | 12.75 (4.37 - 15.12) |  |  | |  |  |
|  | Healthy Liver | 3 | ≤ 8.84 | 11.57 (9.37 - 18.21) | 0.0389 |  | |  |  |
|  |  | 2 | >8.84 | 3.19 (3.02 - 3.35) |  |  | |  |  |

**Supplementary Table V. Sensitivity, specificity, AUC and C-statistic with 95%CI for the Cox survival model in BCLC-C patients attending to circulating miR-518d-5p levels.**

| **Time-points (months)** | **Sensitivity (95%CI)** | **Specificity (95%CI)** | **Area Under the Curve (95%CI)** | **C-statistic Harrell (95%CI)** |
| --- | --- | --- | --- | --- |
|
| **6** | 0.76 (0.62 - 0.91) | 0.63 (0.46 - 0.8) | 0.7 (0.5 - 0.9) | 0.59 (0.52 - 0.66) |
| **12** | 0.55 (0.38 - 0.73) | 0.62 (0.44 - 0.79) | 0.59 (0.41 - 0.76) |  |
| **18** | 0.53 (0.36 - 0.71) | 0.67 (0.5 - 0.84) | 0.6 (0.4 - 0.8) |  |
| **24** | 0.53 (0.35 - 0.7) | 0.78 (0.63 - 0.93) | 0.65 (0.45 - 0.85) |  |

**Supplemental Table VI. List of antibodies.**

| **Antibody** | **Id.** | **Supplier** | **Dilution** | **Incubation Solution** |
| --- | --- | --- | --- | --- |
| **β-ACTIN** | ab8224 | Abcam | 1/10000 | TBST-0.01%-Milk 5% |
| **Β-Catenin** | 9562 | Cell Signaling Technology | 1/1000 | TBST-0.01%-Milk 5% |
| **c-Jun** | 9165 | Cell Signaling Technology | 1/1000 | TBST-0.01%-Milk 5% |
| **GAPDH** | ab8245 | Abcam | 1/10000 | TBST-0.01%-Milk 5% |
| **JNK** | 9252 | Cell Signaling Technology | 1/1000 | TBST-0.01%-Milk 5% |
| **Pan-RAS** | B5494 | LifeSpan Biosciences | 1/2000 | PBS-BSA 0.01% |
| **PCNA** | PCNA (F-2) | Santa Cruz Biotechnology | 1/2000 | PBS-BSA 0.01% |
| **Phospo-c-Jun**  **Ser73** | 3270 | Cell Signaling Technology | 1/1000 | TBST-0.01%-Milk 5% |
| **p44/42 MAPK (Erk1/2)** | 9102 | Cell Signaling Technology | 1/1000 | TBST-0.01%-Milk 5% |
| **Phospho-JNK**  **T183/Y185** | 446826 | Life Technologies | 1/1000 | TBST-0.01%-Milk 5% |
| **Phospho-p44/42 MAPK**  **(Erk1/2) (Thr202/Tyr204)** | 9101 | Cell Signaling Technology | 1/1000 | TBST-0.01%-Milk 5% |
| **Phospho-S6 Ribosomal Protein (Ser235/236)** | #4857S | Cell Signaling Technology | 1/1000 | TBST-0.01%-Milk 5% |
| **P21 (F-5)** | sc-6246 | Santa Cruz Biotechnology | 1/500 | TBST-0.01%-Milk 5% |
| **P65 (C-20)** | Sc-372 | Santa Cruz Biotechnology | 1/1000 | TBST-0.01%-Milk 5% |
| **PUMA** | #4976 | Cell Signaling Technology | 1/1000 | TBST-0.01%-BSA 3% |
| **S6 Ribosomal Protein (54D2)** | #2317S | Cell Signaling Technology | 1/1000 | TBST-0.01%-Milk 5% |
| **HRP-conjugated secondary goat antibody to mouse** | #7076 | Cell Signaling Technology | 1/10000 | TBST-0.01%-Milk 5% |
| **HRP-conjugated secondary goat antibody to rabbit** | #7074 | Cell Signaling Technology | 1/10000 | TBST-0.01%-Milk 5% |
